# Supplementary material for: Mediating role of food insecurity in the relationship between perceived MSM related stigma and depressive symptoms among men who have sex with men in Nepal
Source: PLoS One. 2024 Jan 2;19(1):e0296097. doi: 10.1371/journal.pone.0296097 (PMC10760890; doi:10.1371/journal.pone.0296097)
Supplement: S1 File — (PDF) [file pone.0296097.s001.pdf]

Yale Qualtrics Survey Tool

Consent and Introduction सहमति र परिचय

Study Title: Informing HIV and STI Prevention among men who have sex with men (MSM) in Nepal

Faculty Advisor/Principal Investigator’s Name, Phone Number, E-mail Address:
Local Principal Investigator: Manisha Dhakal, (+977-1- 4443350/4007647), manishadhakal.nepal@gmail.com, Blue Diamond Society

USA Principal Investigator: Roman Shrestha, +1-860-486-2834, roman.shrestha@uconn.edu, University of Connecticut

Study Sponsor: University of Connecticut (UConn)

KEY INFORMATION

The purpose of this form is to provide you information that will help you decide whether or not to voluntarily participate in this research study. Below is some key information to keep in mind when thinking about why you may or may not want to be in the research. The person performing the research will answer any of your questions. Take your time, read the information below, and ask any questions you might have before deciding whether or not to take part. If you decide to be involved in this study, this form will be used to record your consent. You must be at least 18 years of age to participate.

Why is this study being done?

You have been asked to participate in a research study about access and barriers to sexual health care, and sexual health behaviors among MSM in Nepal. The purpose of this study is to explore potential factors influencing human immunodeficiency virus (HIV) and sexually transmitted infections (STI), prevention, and access to sexual health-related care among men who have sex with men (MSM) in Nepal. Data from this study will be used to guide the development of services and interventions to decrease risk for HIV and increase healthy outcomes among MSM in Nepal.

Why am I being asked to take part in this research study?
You are being asked to take part in this study because you were recruited by someone you know who has identified you as eligible for this study, and you can provide valuable information to help improve health services for at-risk MSM in Nepal. Your participation in this study is completely voluntary. You may refuse to participate or stop your participation in this research study at any time without penalty or loss of benefits to you.

How many people will take part in this study?
This study will include approximately 200-250 study participants.

How long will I be in the study?
Your participation will last for about 90 minutes during one visit.

What will happen if I take part in this study?
If you agree to participate in this study, the following will occur:

Survey:

1. If you agree to participate in the study, we will ask you to participate in a survey that will take approximately one hour. The survey will include questions about your demographic characteristics, drug and alcohol use, sexual history and behaviors, experience with healthcare services (general and sexual health related), victimization, social support, mental health, and mobile application/smartphone use.

HIV and Syphilis Testing:

1. If you agree to participate in the study, you will take a free HIV rapid test, and a confirmatory HIV and Syphilis test via a blood draw. If you already know that you are HIV-infected, we would still like to offer you an HIV test today so that we can link today's HIV test result with your survey results. The test will draw 8mL of blood. Your name and other identifying information will not be connected to your blood sample, only your participant ID number will be connected to the blood sample, so it will remain confidential. Once the blood sample is analyzed, it will be destroyed.
2. Linkage: we will link your test results with your survey so we can learn about sexual and drug-use risk behaviors known to be connected with HIV infection. We will link your test results using the same ID assigned to the survey so that your identity remains confidential. This is a confidential test- your name will not be on the test

results or the survey. No one besides you will be told your test results, and neither the survey nor the test will be placed in any medical record.

3. Test Results: within 2-3 days of your blood test, a research staff member will call you to help schedule a time to come back to the research site and pick up your test results.
4. Care Referral: if you test positive for either HIV or Syphilis, we will refer you to the Cruise AIDS Nepal for treatment and counselling if you wish to pursue them.

Can I stop being in this study?
Yes. Your participation is voluntary and you can decide to stop at any time. Just tell the research assistant or staff member conducting the blood draw or survey. It is also your right to choose not to answer any question or any part of a question on the survey if you do not feel comfortable answering it. If you choose to not be in the study or to withdraw from the study it will not affect how you are treated by our research staff or your chance of participating in another research study in the future. Withdrawal or refusing to participate will not affect your relationship with the University of Connecticut or Blue Diamond Society in any way, nor will it affect your ability to get tested for HIV or Syphilis through CruiseAIDS Nepal.
If you withdraw from the study, any data collected up until the point of withdrawal will be stored until study completion and then discarded when the study is over.
Also, the research staff may stop you from taking part in this study at any time if they believe it is in your best interest, if you do not follow the study rules, or if the study is stopped.

What side effects or risks can I expect from being in this study?
The risks involved with participation in this study are low and may include:

1. Phlebotomy: risks of minor injury, such as bruising or infection with venipuncture are unlikely but might still happen. Potential distress due to the blood draw is possible among some participants.
2. Syphilis and HIV testing: there is a risk of potential anxiety over being tested for syphilis and HIV infection. There is a risk that a positive test result for either may cause distress. You will be referred to receive appropriate medical attention should you test positive for any (or both) of these tests. If you wish, you can also be referred to counseling services.
3. Sensitive information: you may feel some psychological discomfort with the sensitive nature of some of the survey questions

4. Confidentiality: steps will be taken to protect the confidentiality of the data and you. Confidentiality will be protected by ensuring that personal identifiers are removed from the data and publications that result from the study and all data will be securely stored with our best efforts to avoid any breaches to confidentiality. Additionally, only the research staff will have access to identifiable information

Are there benefits to taking part in this study?
The benefits of participation are free testing for HIV and syphilis, referral to standard treatment and counseling services provided by Cruise AIDS Nepal in the case of a positive test result, and all participants will be providing valuable and much needed data for that will help improve HIV prevention and treatment services for MSM in Nepal, as well as foster support for the LGBTQIA community.

If I am harmed while participating in the study, who will pay for the necessary medical care?
In the event you become sick or injured during the blood draw, immediately notify the principal investigator or a member of the research team. After informing a member of the research team, you will be offered immediate help (to the best of their ability) and/or referred to medical services as appropriate.
If you require medical care for any psychological distress, we will provide an optional referral to an on-site counselor at the Cruise AIDS Nepal clinic.

Will information about me be kept private?
We will make every effort to protect the confidentiality of study records that identify you, but we cannot guarantee total confidentiality. Survey data collected and results of the blood draw will be uploaded into a secure data warehouse system. All data will be stored in a locked and secure room, computers will be password protected, and only authorized research staff will have access to any data, and your name along with any other identifiable information will be separated from your survey results and blood test results.
Your study information will not be released without your written permission, except as necessary for monitoring by representatives of the study protocol team, UConn Institutional Review Board, and the Nepal Health Research Council’s Institutional Review Committee. If information from this study is published or presented at scientific meetings, your name and other identifiable information will not be used.
Your information will not be used for future research. The information and blood samples that we collected as part of this research, even with identifiers removed, will not be used or

distributed for future research studies.

Will participating in this study cost me anything?
No. There are no direct costs for taking part in this research study, except for your time and/or transportation to the Cruise AIDS Nepal research site.

Will I be paid for taking part in this study?
Yes. You will receive RS 1000 for your time and effort in participating in the blood draw and survey. You will receive RS 500 for each referral that successfully completes participation as well. With the chance to refer up to 5 peers, you can earn up to RS 2500 for referrals. You will receive the payment by cash.

Who can I contact with questions concerning my rights as a research participant?
Prior to, during, or after your participation, you can contact either the University of Connecticut (USA) Institutional Review Board (IRB) Office at +1-860-486-8802 or email irb@uconn.edu, or the Nepal Health Research Council Institutional Review Committee (IRC) at 977-1-4254220 or email nhrc@nhrc.gov.np to:

- Discuss problems, concerns, and questions, including questions about your rights as a person in a research study
- Obtain information
- Offer input.

The IRB and IRC Offices are not affiliated with any specific research study. You can contact them anonymously if you wish.

Who can answer my questions about the study?
Prior to, during, or after your participation, you can contact the Principal Investigators (using the contact information on page one and below) for any questions or concerns or if you feel that you have been harmed or injured as a result of being in the research.
Local Principal Investigator: Manisha Dhakal, (+977-1- 4443350/4007647), manishadhakal.nepal@gmail.com, Blue Diamond Society
USA Principal Investigator: Roman Shrestha, +1-860-486-2834, roman.shrestha@uconn.edu, University of Connecticut

Consenting to be in this Study:
You have been informed about this study’s purpose, procedures, possible risks and

benefits, and you will receive a copy of this form by request. You have been given the opportunity to ask questions before you sign, and you have been told that you can ask other questions at any time. You voluntarily agree to participate in this study.

If you add your electronic signature and click the 'next' button below, you acknowledge that you are at least 18 years of age, that you have read this consent form, and agree to be a participant in this study. By agreeing to participate, you are not waiving any of your legal rights.

× SIGN HERE

clear

Personal details

Name of the participant
सहभागीको नाम

Participant ID
सहभागीको परिचय नम्बर

Socio-demographics

First, we would like to ask some questions about yourself.
सर्वप्रथम, हामी तपाईंको बारेमा केही प्रश्नहरू सोध्न चाहन्छौं।

How old are you? Please enter number in years.
तपाईं कति वर्षको हुनुभयो? कृपया आफ्नो उमेर/ वर्ष नम्बरमा भर्नु होला।

What province were you born in?
तपाईंको जन्म कुन प्रदेशमा भएको हो?

- Province 1 (प्रदेश १)
- Madhesh (मधेस)
- Bagmati (बागमती)
- Gandaki (गण्डकी)
- Lumbini (लुम्बिनी)
- Karnali (कर्णाली)
- Sudurpashchim (सुदूरपश्चिम)

What is your religion?
तपाईंको धर्म के हो?

- Hindu (हिन्दू)
- Buddhist (बौद्ध)
- Muslim (मुस्लिम)
- Christian (क्रिस्चियन)
- ____ Other (specify) (अन्य हो भने खुलाउनु होस):

Which of the following best describes your current sexual orientation?
तलका मध्य तपाईंको हालको यौन अभिमुखिकरण वर्णन कुनले गर्छ?

- Homosexual/gay man (समयौनिक/ समयौनिक पुरुष)
- Bisexual (द्वयौनिक्)
- Straight/heterosexual (विषमयौनिक)
- ____ Other (specify) (अन्य हो भने खुलाउनु होस):

What is the highest level of school or degree you have completed?
तपाईंले पूरा गर्नुभएको उच्च शिक्षाको डिग्री वा स्तर के हो?

- No education (निरक्षर)
- Literate, no formal education (औपचारिक शिक्षा बिना साक्षर)

- School up to 5 class (५ वर्षसम्मको विद्यालय)
- School up to 8 class (६-९ वर्षसम्मको विद्यालय)
- Grade 10 passed (कक्षा १० उत्तीर्ण)
- PCL or +2 passed (प्रवीणता प्रमाणपत्र स्तर वा +२ उत्तीर्ण)
- Graduation and above (स्नातक वा सो भन्दा माथि)

Are you currently employed?
के तपाई हाल रोजगारीमा हुनुहुन्छ?

- No (छैन)
- Yes (छु)

In general, what is your current monthly income in NRs (from all sources)? Please enter just the number.
सामान्यतया, सबै स्रोतहरूबाट तपाईको हालको मासिक आम्दानी कति छ? कृपया नेपाली रुपैयाँ खुलाउनु होस्।

How many people are you supporting with your income now, including yourself? Please enter just the number.
अहिले आफ्नो आम्दानीबाट तपाईं लगायत कति जनालाई सहयोग गरिरहनुभएको छ? कृपया संख्या खुलाउनु होस्।

Which of the following best describes your current intimate relationship status?
तलका मध्ये कुनले तपाईको हालको घनिष्ठ सम्बन्धको स्थितिलाई राम्रोसँग वर्णन गर्दछ?

- Single (अविवाहित)
- Have a boyfriend/partner (एक प्रेमी/पार्टनर भएको)
- Legally married (कानुनी रूपमा विवाह भएको)
- Widowed (विधवा/विधुर)
- Divorced or separated from spouse (सम्बन्धविच्छेद भएको वा जीवनसाथीबाट अलग भएको)

Which of the following best describes your current living situation?
तलका मध्ये कुनले तपाईको हालको जीवन अवस्थालाई राम्रोसँग वर्णन गर्दछ?

- Homeless (living on the street) {घरबारविहीन (सडकमा बस्ने)}
- Living in own home (आफ्नै घरमा बस्ने)
- Living in residential hotel (आवासीय होटेलमा बस्ने)
- Rented apartment/room (भाडाको अपार्टमेन्ट/कोठा बस्ने)
- [ ] Other (specify) (अन्य हो भने खुलाउनु होस):

During the last 12 months, was there a time when, because of lack of money or other resources:
विगत १२ महिनामा, पैसा वा अन्य स्रोतको अभावले गर्दा यस्तो समय आएको थियो:

	No (थिएन)	Yes (थियो)
You were worried you would not have enough food to eat? (के तपाईं प्रयाप्त खाना छैन भनेर चिन्तित हुनुहुन्थ्यो?)	○	○
You were unable to eat healthy and nutritious food? (के तपाईं स्वस्थ र पौष्टिक खाना खान असमर्थ हुनुहुन्थ्यो?)	○	○
You ate only a few kinds of foods? (के तपाईंले केही प्रकारका खानेकुरा मात्र खानुहुन्थ्यो?)	○	○
You had to skip a meal? (के तपाईंले खानाखान छोड्नुहुन्थ्यो?)	○	○
You ate less than you thought you should? (के तपाईंले खाना खानपर्ने भन्दा कम खानुहुन्थ्यो?)	○	○
Your household ran out of food? (के तपाईको घरको खाना सकिनथ्यो ?)	○	○
You were hungry but did not eat? (के तपाईंले भोक लगेको बेलामा खानाखान पाउनु भएन?)	○	○
You went without eating for a full day? (के तपाईंले दिन भर खाना खान पाउनुभएन ?)	○	○

Drug, Alcohol Use, Smoking and Tobacco use

Now, we are going to ask you some questions about drug or alcohol use. Please try to answer as honestly as possible, and remember that your answers are completely anonymous.

अब हामी तपाईलाई लागूऔषध र मदिराको प्रयोग बारेमा प्रश्नहरु सोध्ने छौ| तपाईको जबाफहरु पूर्ण रुपले गोप्य हुने छन् त्यसैले ढुक्क भएर सहि उत्तर दिनुहोस्|

Below is a list of substances (alcohol and drugs). Have you ever used any of these even once in your life? Select all that apply.
तल लागूऔषध प्रयोग, मदिराको प्रयोग जस्ता पदार्थको नाम दिईएको छ| के तपाईले जीबनमा कुनै पनि पदार्थको प्रयोग गर्नु भएको छ ? छ भने मिल्ने जति सबै चयन गर्नुहोस्|

- ☐ **Alcohol** (मदिरा वा रक्सी सेवन)
- ☐ **Tobacco** (सुर्ती जन्य पदार्थको प्रयोग)
- ☐ Smoking Tobacco (चुरोट, हुक्का, भपे, इ-सिग्रेट)
- ☐ **Cannabis**, Hashish, Weed, Marijuana (चरस, गाजा, भाङ, धतुरो)
- ☐ **Basecoke**, Crack
- ☐ **Cocaine**, Coke, Charlie, Blow, Snow, White
- ☐ **Crystal meth**, Tina, Ice, Crystal, T, Shabu, Yaba, Shista, Glass
- ☐ **GHB** or **GBL**, G, Liquid Ecstasy, Gina
- ☐ **Heroin**, Smack, H, Brown, Sugar, Chiva, Chiba
- ☐ **Ketamine**, K, Special K, Keta, Ket, Vitamin K, Donkey Dust
- ☐ **Laughing gas**, Nitrous Oxide
- ☐ **LSD**, Acid
- ☐ **Mephedrone**, Meow Meow, 4-MCC, M-Cat, Miaow, Plant food, MMC-4, Bubble
- ☐ **XTC** or **MDMA**, M, Molly, Ecstasy, Pill, Candy
- ☐ **MXE**, Methoxet amine, Mexxxy, Roflocptr
- ☐ **Naphyrone**, NRG
- ☐ **Magic mushrooms**
- ☐ **Poppers**, Nitrite Inhalants
- ☐ **Ritalin**, Concerta, Dexamphetamine (not used as medication for ADD/ADHD)
- ☐ **Speed**, Amphetamine, Pep
- ☐ **Designer drugs** (2-CB, 3-MMC, 4-FA, 4-MEC)
- ☐ None

In the past 6 months, which substances have you used? Select all that apply.
विगत ६ महिनामा तपाईले कुन लागूऔषध प्रयोग गर्नुभयो? लागू हुने सबै चयन गर्नुहोस्।

- ☐ » **Alcohol** (मदिरा वा रक्सी सेवन)

- ☐ » **Tobacco** (सुर्ती जन्य पदार्थको प्रयोग)
- ☐ » Smoking Tobacco (चुरोट, हुक्का, भपे, इ-सिग्रेट)
- ☐ » **Cannabis**, Hashish, Weed, Marijuana (चरस, गाजा, भाङ, धतुरो)
- ☐ » **Basecoke**, Crack
- ☐ » **Cocaine**, Coke, Charlie, Blow, Snow, White
- ☐ » **Crystal meth**, Tina, Ice, Crystal, T, Shabu, Yaba, Shista, Glass
- ☐ » **GHB** or **GBL**, G, Liquid Ecstasy, Gina
- ☐ » **Heroin**, Smack, H, Brown, Sugar, Chiva, Chiba
- ☐ » **Ketamine**, K, Special K, Keta, Ket, Vitamin K, Donkey Dust
- ☐ » **Laughing gas**, Nitrous Oxide
- ☐ » **LSD**, Acid
- ☐ » **Mephedrone**, Meow Meow, 4-MCC, M-Cat, Miaow, Plant food, MMC-4, Bubble
- ☐ » **XTC** or **MDMA**, M, Molly, Ecstasy, Pill, Candy
- ☐ » **MXE**, Methoxet amine, Mexxxy, Roflocptr
- ☐ » **Naphyrone**, NRG
- ☐ » **Magic mushrooms**
- ☐ » **Poppers**, Nitrite Inhalants
- ☐ » **Ritalin**, Concerta, Dexamphetamine (not used as medication for ADD/ADHD)
- ☐ » **Speed**, Amphetamine, Pep
- ☐ » **Designer drugs** (2-CB, 3-MMC, 4-FA, 4-MEC)
- ☐ None

In the past 6 months, have you used more than one of these substances on the same day?
बिगतको ६ महिनामा, के तपाईले एक भन्दा धेरै पदार्थको सेवन एकै दिन गर्नु भएको छ?

- ○ No (छैन)
- ○ Yes (छ)

Have you ever drank alcohol?
के तपाईले जीबनमा कहिलै मदिरापान गर्नु भएको छ?

- ○ No (छैन)
- ○ Yes (छ)

Do you currently drink alcohol (in the past 12 months)?
तपाईले हाल मदिरापान सेवन गर्नुहुन्छ? (विगत १२ महिनामा)

- No (छैन)
- Yes (छ)

How many drinks did you have on a typical day when you were drinking in the past year?
विगत बर्षमा, तपाईले मदिरा (रक्सी) सेवन गरेको बेला, एक दिनमा प्रायजसो कति ड्रिङ्कस खानुहुन्थ्यो?

- 1 or 2 (१ वा २)
- 3 or 4 (३ वा ४)
- 5 or 6 (५ वा ६)
- 7, 8, or 9 (७, ८, वा ९)
- 10 or more (१० वा धेरै)
- Not applicable (लागु हुदैन)

How often did you have six or more drinks on one occasion in the past year?
विगत ६ महिनामा, कति पटक तपाईले कम्तिमा ६ वा धेरै ड्रिंक मदिरा (रक्सी) सेवन गर्नुभयो?

- Never (कहिलै पनि गरिन)
- Less than monthly (एक महिना भन्दा थोरै)
- Monthly (मासिक)
- Weekly (हप्ताहिक)
- Daily or almost daily (दैनिक वा दैनिक जस्तो)

Have you ever injected drugs?
के तपाईले जीबनमा कहिलै लागूऔषधको सेवन सिरिन्ज वा सुई देखि गर्नु भएको छ?

- No (छैन)
- Yes (छ)

In the past 6 months, have you injected drugs?
बिगत ६ महिनामा, के तपाईले लागूऔषधको सेवन सिरिन्ज वा सुई देखि गर्नु भएको छ?

- No (छैन)
- Yes (छ)

How old were you when you first injected drugs? Please enter in just the number.
तपाईले पहिलो चोटी सिरिन्ज वा सुई देखि लागूऔषधको सेवन गर्ने प्रयास गर्दा कति उमेरको हुनु हुन्थ्यो?

Have you ever tried smoking tobacco like cigarette, hukka, e-cigarette and vape?
तपाईले कहिल्यै कुनै सुर्तिजन्य धुम्रपान सेवन गर्नुहुन्छ? (जस्तै बिडी, चुरोट, तमाखु, हुक्का वा अन्य स्थानिय रुपमा उत्पादन हुने सुर्तितजन्य पदार्थहरु)

- No (गर्दिन)
- Yes (गर्छु)

Do you currently smoke daily?
के अहिले दैनिक धुम्रपान गर्नुहुन्छ?

- No (गर्दिन)
- Yes (गर्छु)

At what age did you start smoking?
पहिलो पटक धुम्रपान गर्न शुरु गर्दा तपाईको उमेर कति वर्षको थियो?

Have you ever tried smokeless tobacco like like (Tobacco, Betel Quid, Pan Masala, Chewing Tobacco etc.)?
तपाईले कहिल्यै धुँवारहित सूर्ति जस्तै (सुर्ति, बेटेल, गुट्का, पान मसला, खैनी आदि) को सेवन गर्नुभएको छ?

- No (छैन)
- Yes (छ)

Do you currently use any smokeless tobacco products (In the past 12 months)?
तपाईले हाल कुनै धुँवारहित सूर्तिजन्य पदार्थ सेवन गर्नुहुन्छ? (विगत १२ महिनामा)

- No (गर्दिन)
- Yes (गर्छु)

How old were you when you first tried smokeless tobacco?
पहिलो पटक धुँवारहित सूर्तिजन्य पदार्थ सेवन गर्दा तपाईको उमेर कति वर्षको थियो?

Sexual Behaviors & Sexual History

The following questions will ask about engagement in sexual activities. Your answers are completely anonymous. Please answer them as truthfully as you can.
अब हामी तपाईलाई यौन क्रियाकलाप बारेमा प्रश्न सोध्नेछौं| तपाईंका जवाफहरू पूर्ण रूपमा गोप्य रहनेछन। कृपया तिनीहरूलाई सक्दो सत्यताका साथ जवाफ दिनुहोल।

Have you ever had sexual intercourse? I mean any type of anal and/or vaginal/oral sex even if you were forced to have it.
के तपाईले जीबनमा यौन सम्पर्क (गुदा, मौखिक सेक्स, अथवा यौनि) गर्नु भएको छ?

- No (छैन)
- Yes (छ)

At what age did you first have sexual intercourse? I mean any type of anal, oral, and/or vaginal sex even if you were forced to have it. Please enter in just the number.
तपाईले पहिलो चोटी यौन सम्पर्क गर्दा कति बर्षको हुनु हुन्थ्यो? (कुनै पनि प्रकारको सम्पर्क गुद वा यौन वा मौखिक सेक्स कसैले कर गरेर)|

In the past 6 months, have you had anal sex with another man?
बिगतको ६ महिनामा के तपाईले कोहि पुरुष संग गुदा यौन सम्पर्क गर्नु भएको थियो?

- No (छैन)
- Yes (छ)

In the past 6 months, with how many different men have you had anal sex? Please enter in just the number.

बिगतको ६ महिनामा तपाईले कति जना फरक पुरुष संग गुदा यौन सम्पर्क गर्नु भएको छ?

In the past 6 months, how often did you use a condom during anal sex?
बिगत ६ महिनामा के तपाईले पुरूष संग गुदा यौन सम्पर्क गर्दा कण्डम प्रयोग गर्नु भएको छ?

- Never (कहिलै पनि छैन)
- Rarely (बिरलै)
- Sometimes (कहिले काहिँ)
- Always (सधै)

In the past 6 months, have you had sex with someone living with HIV?
बिगतको ६ महिना के तपाईले कोहि एचआईभी भएको व्यक्ति संग यौन सम्पर्क गर्नु भएको छ?

- No (छैन)
- Yes (छ)
- Not sure (थाहा छैन)

Is your HIV-positive sex partner's HIV viral load "undetectable"?
के तपाईको एचआईभी संक्रमित पाटनरको एच. आइ.भी. भाइरल लोड पत्ता लगाउन नसकिने थियो?

- No (थिएन)
- Yes (थियो)
- Not sure (थाहा भएन)

In the past 6 months, have you had sex with someone who injects drugs?
बिगत ६ महिनामा के तपाईले सुई देखि लागूऔषध सेवन गर्ने व्यक्ति संग यौन सम्पर्क गर्नु भएको थियो?

- No (छैन)
- Yes (छ)
- I do not know (थाहा छैन)

In the past 6 months, have you participated in group sex (having sex with more than 1 man at the same time)?

बिगत ६ महिनामा के तपाई सामुहिक यौन सम्पर्क (एक व्यक्ति भन्दा धेरै पुरुष संग एकै चोटी यौन सम्पर्क) गर्नु भएको छ?

- No (छैन)
- Yes (छ)
- I do not know (थाहा छैन)

Have you ever engaged in sex work? By that, I mean exchanging sex for money, goods, or a place to stay.
के तपाई कहिलै यौनजन्य क्रियाकलाप (पैसा वा कुनै उपहार, समानको लागि यौन सम्पर्क) गर्नु भएको छ?

- No (छैन)
- Yes (छ)

In the past 6 months, have you engaged in sex work?
बिगत ६ महिनामा के तपाई यौनजन्य क्रियाकलापहरुमा संलग्न हुनु भएको छ?

- No (छैन)
- Yes (छ)

How often do you use a condom when you have sex with a client?
तपाईले ग्राहकहरुसंग यौन सम्पर्क गर्दा कण्डमको प्रयोग कत्तिको गर्नु हुन्छ?

- Never (कहिलै पनि गर्दिन)
- Rarely (बिरलै)
- Sometimes (कहिले कहिलै)
- Always (सधै)

What type of sex work or work in the sex industry have you ever done? Select all that apply.
तपाई तल मध्य कस्तो यौनजन्य क्रियाकलापमा सहभागी हुनु भएको छ? मिल्ने जति सबै चयन गर्नु होस्|

- ☐ Street-based sex work (सडकमा आधारित यौन कार्य)
- ☐ Sex work advertised online (अनलाईन देखि गर्ने यौन कार्य)
- ☐ Sex work advertised in magazines or newspapers (पत्रिका वा पत्रपत्रिकाहरूमा विज्ञापन देखि गर्ने यौन कार्य)

- ☐ Informal sex work through word of mouth, occasional hook ups with dates in my networks, or things like that (अनौपचारिक यौन कार्य जस्तै मेरो नेटवर्कमा भएक साथी संग कहिले काँही हुक अपहरू, वा त्यस्ता चीजहरू देखि बोलेर)
- ☐ Escort/rent boy with an agency (कोहि एजेन्ट देखि कोहि केटा भाडामा बोलाएर)
- ☐ Pornography/picture or video (पोर्नोग्राफी/फोटो वा भिडियो)
- ☐ Phone sex (फोन सेक्स)
- ☐ Webcam work (वेबक्याम देखि गरिने यौनजन्य क्रियाकलाप)
- ☐ Erotic dancer/stripper (कामुक नृत्य/ स्ट्रिपर)
- ☐ ________ Other (specify):(अन्य भए खुलाउनुहोस्)

How many years have you been doing sex work? Please enter just the number.
तपाईले कति बर्ष देखि यौनजन्य क्रियाकलापहरु गर्दै आउनु भएको छ? (बर्षमा खुलाउनुहोस्)

____

How many clients do you have sex with in a day on average? Please enter just the number.
औसतमा तपाईले एक दिनमा कति जना ग्राहक संग यौनसम्पर्क गर्नु हुन्छ ? (संख्या खुलाउनुहोस्)

____

How do you find clients? Select all that apply.
तपाईले ग्राहकहरु कसरी पाउनु हुन्छ? (मिल्ने जति सबैमा चिन्ह लगाउनु होस्)

- ☐ Online (अनलाईन)
- ☐ Dating app (डेटिङ एप)
- ☐ Brothel (वेश्यालय)
- ☐ Street/park/cruising area (सडक /पार्क)
- ☐ ________ Other (specify): (अन्य भए खुलाउनुहोस्)

"Chem fun or Chem sex" is the use of ecstasy (MDMA, MDA, Molly), psychedelics (LSD, Foxy), crystal methamphetamine (Meth, ice, Syabu, Pil Kuda), Ketamine, GHB/GBL before or during sex.
"केम फन वा केम सेक्स" भनेको एक्स्टसी (MDMA, MDA, Molly), साइकेडेलिक्स (LSD, Foxy), क्रिस्टल मेथाम्फेटामाइन (Meth, ice, Syabu, Pil Kuda), Ketamine, GHB/GBL सेक्स गर्नु अघि वा सेक्स गर्ने समयमा प्रयोग गर्नु हो।

Have you ever engaged in chemsex even just once?
के तपाईले कहिलै केम सेक्स गर्नु भएको छ?

- No (छैन)
- Yes (छ)

In the past 6 months, have you had chemsex even just once?
बिगतको ६ महिनामा, के तपाईले कहिलै केम सेक्स गर्नु भएको छ?

- No (छैन)
- Yes (छ)

Which of the following drugs have you used during chem fun or chem sex? Select all that apply.
केम सेक्स वा केम फन गर्दा तपाईले तल दिएको कुन ड्रग्स प्रयोग गर्नु भएको छ? (मिल्ने जति सबै चयन गर्नु होस्)

- Ecstasy (MDMA, MDA, Molly)
- Psychedelics (e.g. LSD, Foxy, etc.)
- Crystal Methamphetamine (Meth, Ice, Syabu, PilKuda,etc.
- Ketamine (Vitamin K, Special K)
- GHB, GBL
- ________ Other (specify):

At what age did you first engage in chem fun or chemsex? Please enter just the number.
तपाई पहिलो पटक केम सेक्स गर्दा कति बर्षको हुनु हुन्थ्यो?

____

How often do you participate in chemsex?
तपाई केम सेक्स कति पटक गर्नु हुन्छ?

- 2-3 times a week (हप्ताको २ वा ३ चोटी)
- Once a week (हप्तामा एक चोटी)
- Once a month (महिनामा एक चोटी)
- Once every 6 months (हरेक ६ महिनामा)

- Once every year (हरेक बर्षमा)

Have you ever considered seeking or sought professional help or support related to chemsex?
के तपाईंले कहिल्यै केमसेक्ससँग सम्बन्धित परामर्शदाता संग मद्दत वा समर्थन खोज्नु भएको वा खोज्ने सोच गराउनु भएको छ?

- No (छैन)
- Yes (छ)

HIV/STI Testing Practices

Now, we would to ask a bit about HIV and some related aspects. I want to re-emphasize that your responses are completely anonymous, and we appreciate you answering as truthfully as possible.
हामी अब तपाईलाई एचआईभी सम्बन्धि प्रश्नहरु सोध्ने छौ| हामी पुन पनि तपाईलाई तपाईले दिएका जानकारीहरु गोप्य राखिने छ त्यसैले सकेसम्म साचो कुरा भन्नु हुन अनुरोध गर्दछौ|

Have you ever been tested for HIV?
के तपाईले कहिलै एचआईभीको जाच गर्नु भएको छ?

- No (छैन)
- Yes (छ)

When did you last test for HIV? Please enter the date in DD/MM/YYYY format in AD (put 01 if you are unsure about the exact day).
तपाईले अन्तिम पटक एचआईभीको परिक्षण कहिले गर्नु भएको थियो? पूर्ण मिति लेख्नु होस् (यदि थाहा नभए ०१ लेख्नु होस्)

When did you last test for HIV? Please enter the date in DD/MM/YYYY format in AD (put 01 if you are unsure about the exact day).
तपाईले अन्तिम पटक एचआईभीको परिक्षण कहिले गर्नु भएको थियो? पूर्ण मिति लेख्नु होस् (यदि थाहा नभए ०१ लेख्नु होस्)

Where did you have your most recent test for HIV?
तपाईले हालसालै एचआईभी परिक्षण कहाँ गर्नु भयो?

- NGO/Community-based organization (सामुदायिक संस्था मा /एनजीओमा)
- Government clinic/hospital (सरकारी अस्पतालमा)
- Private Clininc/Hospital (निजि अस्पतालमा)
- Pathlab/private lab (निजि ल्याबमा वा प्याथ ल्याबमा)
- Self-test at home (घरमा आफै)
- Other (specify): (अन्य भए खुलाउनुहोस्)

What was your last HIV test result?
तपाईको अन्तिम पटकको एचआईभी परिक्षणको नतिजा के थियो?

- Negative (नेगेटिभ)
- Positive (पोजेटिभ)
- Don't know (थाहा भएन)

How long have you been living with HIV (months)?
तपाई एचआईभी संक्रमित हुनु भएको कति समय भयो?

Have you sought medical care for HIV?
के तपाईंले एचआईभीको लागि चिकित्सा हेरचाह खोज्नुभएको छ?

- No (छैन)
- Yes (छ)

Are you currently taking ART to treat HIV?
के तपाईँ हाल एचआईभी उपचार गर्न ART लिइरहनुभएको छ?

- No (छैन)
- Yes (छ)

In the last 30 days, how do you rate your compliance to taking ART as prescribed? 0 = I missed all of my doses, and 30 = I missed none of my doses.
बिगतको 30 दिनमा, तपाइँ ART लाई तोकिएबमोजिम सेवन गर्ने लाई कसरी मूल्याङ्कन गर्नुहुन्छ? 0 = मैले सबै मात्रा छुटाए ३० = मैले कुनै पनि मात्रा छुटाएको छैन

	0	3	6	9	12	15	18	21	24	27	30	
ART Compliance (ART तोकिएबमोजिम सेवन)												☐

Have you had a HIV viral load test?
के तपाईंले एचआईभी भाइरल लोड परीक्षण गर्नुभएको छ?

- ○ No (छैन)
- ○ Yes (छ)
- ○ I do not know (थाहा छैन)

In your most recent HIV viral load test, were you virally suppressed or undetectable?
तपाईंको सबैभन्दा हालको एचआईभी भाइरल लोड परीक्षणमा, के तपाईंको भाइरल दमन भएको थियो वा पत्ता लगाउन नसकिने थियो?

- ○ Yes, I was virally suppressed or had an undetectable viral load (हो, मेरो भाइरल लोडमा दमन थियो वा पत्ता लगाउन नसकिने भाइरल लोड थियो।)
- ○ No, I was not virally suppressed or had a detectable viral load (हो, मेरो भाइरल लोडमा दमन थिएन वा पत्ता लगाउन सकिने भाइरल लोड भएको थिएन)
- ○ I do not know (थाहा छैन)

Have you disclosed your HIV status to any of the following:
के तपाईंले आफ्नो एचआईभी स्थिति निम्न मध्ये कुनैमा खुलासा गर्नुभएको छ:

- ☐ Family members (परिवारका सदस्य)
- ☐ Friends (साथीहरू)
- ☐ At workplace (कार्यस्थलमा)
- ☐ Healthcare Provider (स्वास्थ्य सेवा प्रदायक)
- ☐ [ ] Other (specify): (अन्य भए खुलाउनुहोस्)

Have you ever been discriminated against by health workers because of your HIV status?
के तपाईलाई तपाईको एचआईभी स्थितिका कारण स्वास्थ्यकर्मीहरूद्वारा भेदभाव गरिएको छ?

- ○ No (छैन)
- ○ Yes (छ)

What do you think is your current risk of getting HIV?
तपाईलाई अहिले HIV लाग्ने जोखिम कति छ जस्तो लाग्छ?

- ○ None (छैन)
- ○ Low (थोरै)
- ○ Moderate (ठिकै)
- ○ High (धेरै)

Have you ever been diagnosed with any of the following sexually transmitted infections? Select all that apply.
के तपाईलाई अहिले सम्म तल मध्ये कुनै यौन सम्पर्क देखि सर्ने संक्रमण निदान भएको छ? लागू हुने सबै चयन गर्नुहोस्।

	No (छैन)	Yes (छ)
Syphilis (सिपिलिस / भिरिंगी)	○	○
Gonorrhea (गोनोरिया)	○	○
Chlamydia (क्लामिडिया)	○	○
Other (specify): (अन्य भए खुलाउनुहोस्) [ ]	○	○

In the past 6 months, have you been diagnosed with any of the following sexually transmitted infections? Select all that apply.
बिगत ६ महिनामा, के तपाईलाई यौन सम्पर्क देखि सर्ने रोगको निदान भएको थियो? लागू हुने सबै चयन गर्नुहोस्।

- ☐ » Syphilis (सिपिलिस / भिरिंगी)
- ☐ » Gonorrhea (गोनोरिया)
- ☐ » Chlamydia (क्लामिडिया)

- ☐ [ ] » Other (specify): (अन्य भए खुलाउनुहोस्)

Before participating in this survey, have you ever heard of HIV self-testing at home?
(तपाईले यस अध्यनमा सहभागी हुनु भन्दा पहिला कहिलै घरमै एचआईभी परिक्षण गरिन्छ भनेर सुन्नु भएको थियो?)

- ○ No (थिएन)
- ○ Yes (थियो)

Have you ever used a HIV self-testing kit?
के तपाईले अहिले सम्म आफ्नो एचआईभीको परिक्षण आफै HIV Self Testing किट प्रयोग गरेर गर्नु भएको छ?

- ○ No (छैन)
- ○ Yes (छ)

HIV self-testing is a process in which a person collects his own specimen (oral fluid or blood) using a simple rapid HIV test and then performs the test and interprets the result, often in a private setting, either alone or with someone they trust.
एचआईभीको आफै परिक्षण गर्ने भनेको कुनै व्यक्तिले आफ्नै नमुना (मुख देखि तरल पदार्थ वा रगत) निकालेर एचआईभी परिक्षण गरेर आफु संग मिल्ने साथी वा विश्वास गर्ने व्यक्ति संग बसेर घरमै बसेर नतिजा हेर्न सकिन्छ|

If you could take an HIV test at home, would you be willing to use an HIV self-testing kit?
यदि तपाईले HIV Self Testing किट घरमा प्रयोग गर्न मिल्छ भने, के तपाईले सो किट प्रयोग गर्न इच्छुक हुनुहुन्छ?

- ○ No (गर्दिन)
- ○ Yes (गर्छु)

What type of specimen do you prefer to use for HIV self-testing at home?
घरमा HIV Self Testing गर्दा तपाईले तल मध्य कुन नमुना संकलन गर्न रुचाउनु हुन्छ?

- ○ Blood-based (finger stick/prick) {रगतमा आधारित (औंला देखि निकालेर)}
- ○ Oral fluid based (saliva) (मुख देखि तरल पदार्थ निकालेर)

- ◯ Urine-based (पिसाबमा आधारित)

Where would you prefer to get the HIV self-testing kit from? Select all that apply.
HIV Self Testing किट तपाईले कहाँ देखि लिन रुचाउनुहुन्छ? तल मिल्ने जति सबैमा टिक लगाउनुहोस्|

- ☐ Retail pharmacy (फार्मेसी देखि)
- ☐ Convenience Stores (मार्ट देखि)
- ☐ Online shopping site (website/app) {अनलाइन किनमेल साइट (वेबसाइट/एप) देखि}
- ☐ NGO/ Community-based organization (एन जी वो / सामुदायिक संस्था)
- ☐ Government Clinic/Hospital (सरकारी अस्पताल)
- ☐ Private Clinic/Hospital (निजि अस्पताल)
- ☐ ____________ Other (specify): (अन्य भए खुलाउनुहोस्)

How would you like to receive pre and post-test counseling? Select all that apply.
तपाईले टेस्ट गर्नु अघि र पछिको परामर्श कसरी लिन चाहनुहुन्छ? तल मिल्ने जति सबैमा टिक लगाउनुहोस्|

- ☐ Virtual consultations (e.g. Video calls, Zoom) {भर्चुअल परामर्श (भिडियो कल, जुम)}
- ☐ Online Videos (अनलाइन भिडियो)
- ☐ Face-to-face consultation (आमनेसामने भएर परामर्श)
- ☐ Telephone Hotlines (टेलीफोन हटलाईन)
- ☐ Written leaflets (लिखित पर्चाहरू)
- ☐ Mobile Apps (मोबाइल एपहरू)
- ☐ WhatsApp/ WeChat/ Instant messaging/SMS (व्हाट्सएप/ WeChat/ तत्काल सन्देश/SMS)
- ☐ ____________ Other (specify): (अन्य भए खुलाउनुहोस्)

What is the maximum price you are willing to pay for an HIV self-test?
एचआईभी सेल्फ टेस्टिंगको लागि अधिकतम् कति सम्म पैसा तिर्न सक्नु हुन्छ?

- ◯ NRs 100-500 (नेरु १००-५००)
- ◯ NRs 501-1000 (नेरु ५०१ - १०००)
- ◯ NRs 1001-1500 (नेरु १००१ - १५००)
- ◯ More than NRs 1500 (नेरु १५०० भन्दा धेरै)

What do you perceive are some of the benefits of HIV self-testing? Select all that apply.
तपाईलाई एचआईभी सेल्फ टेस्टिंगको केहि फाईदाहरु के के हुन् लाग्छ? तल मिल्ने जति सबैमा टिक लगाउनुहोस्|

- ☐ HIV Self-testing is convenient (e.g. I can avoid travel time and can test on my own schedule) {एचआईभी सेल्फ टेस्टिंग धेरै सहज हुने (मैले हिड्ने समय खर्च गर्नु पर्दैन र मेरो आफ्नै समयमा टेस्ट गर्न मिल्ने भएर)}
- ☐ Offers privacy as I can do a self-test alone or with someone I trust {गोपनीयताको हुने (तपाईले एक्लै वा तपाईले विश्वास गर्ने व्यक्ति संग बसेर गर्न सक्नु हुन्छ)}
- ☐ Offers quick results (नतिजा छिटो आउने)
- ☐ Do not need to go to clinic in person (कुनै पनि क्लिनिक वा स्वास्थ्य संस्था जानु पर्दैन)
- ☐ I do not think HIV self-testing has any benefit (एचआईभी सेल्फ टेस्टिंगको कुनै पनि फाईदा छैन)

What concerns you about HIV self-testing? Select all that apply.
तपाईलाई एचआईभी सेल्फ टेस्टिंगको बारेमा के के चिन्ता छ? लागू हुने सबै कुरा चयन गर्नुहोस्।

- ☐ I do not know where to get the test kit (मलाई टेस्ट किट कहाँ पाहिन्छ भनेर थाहा नभएर)
- ☐ I am afraid of positive result (मलाई नतिजा पोजेटिभ आउन सक्छ भनेर डर लाग्छ)
- ☐ I am afraid I might use the test kit wrong (मलाई गलत परिक्षण सामग्री प्रयोग गर्छु कि भनेर डर लाग्छ)
- ☐ I think the Kit is difficult to use (मलाई किट प्रयोग गर्न गार्हो हुन्छ जस्तो लाग्छ)
- ☐ I think the results are difficult to interpret (मलाई नतिजाको व्याख्या गर्न गाह्रो हुन्छ जस्तो लाग्छ)
- ☐ I am concerned about the accuracy and quality of the test kit (म टेस्ट किटको गुणस्तर र यथार्थताको बारेमा चिन्तित छु)
- ☐ HIV self-testing kit is not affordable (एचआईभी सेल्फ टेस्टिंग किट किन्न नसकेर)
- ☐ If the result is positive, I do not know where to go for confirmatory testing (मलाई परिक्षणको नतिजा पोजेटिभ आयो भने नतिजा पुस्टि गर्न कहाँ जाने भनेर थाहा नभएर)
- ☐ I will need counseling support, which may not be available (मलाई परामर्श सहयोग चाहिन्छ तर त्यो उपलब्ध छैन)
- ☐ I use self-testing at home (मैले सेल्फ टेस्टिंग घरमै प्रयोग गर्छु)
- ☐ Nothing concerns me about HIV self-testing (मलाई एचआईभी सेल्फ टेस्टिंगको बारेमा कुनै पनि चिन्ता छैन)

Pre-Exposure Prophylaxis (PrEP) सम्पर्क अधिको प्रोफिल्याक्सिस

Before participating in this survey, have you ever heard about PrEP, also known as Pre-exposure Prophylaxis?
यो अध्यनमा सहभागी हुनु भन्दा पहिला के तपाईले सम्पर्क अघिको प्रोफिल्याक्लिसस बारेमा सुन्नु भएको छ?

- No (छैन)
- Yes (छ)

PrEP is an HIV-prevention medication for HIV-negative people. In Nepal, the medication is most commonly sold under the brand names of Tenvir-EM or Tenof-EM. PrEP can prevent someone from becoming HIV-infected, even when they are exposed to HIV through high-risk activities such as sex without using a condom or injecting drug use. PrEP is approved by the World Health Organization and Ministry of Health.
सम्पर्क अघिको प्रोफिल्याक्लिससको भनेको एचआईभी देखि बच्नलाई एचआईभी नलागेका ब्यक्तिहरुले सेवन गर्ने औषधि हो | यो नेपालमा सामान्यतया टेनभिर -ईयम अथवा टेनोफ - ईयम को नाममा खरिद बिक्रि गरिन्छ| सम्पर्क अघिको प्रोफिल्याक्लिससले एचआईभी संक्रमणको अति जोखिममा भएका व्यक्तिहरु जस्तै बिना कण्डम यौन सम्पर्क गर्ने , लागूऔषधको सेवन गर्ने (सुई) लाई रोग लाग्न देखि बचाऊ छ| यो स्वास्थ्य मन्त्रालय र बिश्वा स्वास्थ्य संगठनले पनि स्वीकृत दिएको छ|

Have you ever used PrEP?
के तपाईले अहिलेसम्म सम्पर्क अघिको प्रोफिल्याक्लिसस प्रयोग गर्नु भएको छ?

- No (छैन)
- Yes (छ)

Are you currently taking PrEP?
के तपाईले अहिले सम्पर्क अघिको प्रोफिल्याक्लिसस प्रयोग गर्नु भएको छ?

- No (छैन)
- Yes (छ)

Which of the following best describes the way that you are taking PrEP?
तल मध्य तपाईलाई कुनले राम्रो संग सम्पर्क अघिको प्रोफिल्याक्लिसस बारेमा चिनाउछ?

- Single pill daily (दैनिक एक मात्रा पिल)
- On-Demand (2 tablets before sex, 1 tablet each for the next 2 days post sex) {माग अनुसार (सम्पर्क अघि २ वटा ट्याब्लेट,वा सम्पर्क पछि १ वा २ वटा ट्याब्लेट)}

- ☐ Other (specify): (अन्य भए खुलाउनुहोस्)

In the last 30 days, how do you rate your compliance to taking PrEP as prescribed?
0 = I missed all of my doses and 30 = I missed none of my doses.
बिगतको 30 दिनमा, तपाइँ PrEP लाई तोकिएबमोजिम सेवन गर्ने लाई कसरी मूल्याङ्कन गर्नुहुन्छ?
0 = मैले सबै मात्रा छुटाए ३० = मैले कुनै पनि मात्रा छुटाएको छैन

	0	3	6	9	12	15	18	21	24	27	30	
PrEP Compliance (PrEP को पालना)												☐

Where do you go for your PrEP care?
तपाई PrEP care को लागि कहाँ जानु हुन्छ?

- Government clinic/hospital (सरकारी अस्पताल)
- Private Clinic/hospital (निजि अस्पताल)
- Community-based clinic (सामुदायिक स्वास्थ्य चौकी)
- Online (अनलाईन)
- Travel to another country (म अर्को देश जान्छु)
- ☐ Other (specify): (अन्य भए खुलाउनु होस्)

Since you started using PrEP, has your number of sexual partners or sexual contacts:
तपाईले PrEP प्रयोग गर्न थाले पछि यौन सम्पर्क गर्ने साथी र सम्पर्कको संख्यामा कस्तो असर पर्यो?

- Decreased (घट्यो)
- Stayed about the same (उस्तै भयो)
- Increased (बढ्यो)

Since you started using PrEP has your condom use:
PrEP को प्रयोग गर्न थाले पछि कण्डमको प्रयोग मा कस्तो असर पर्यो?

- Decreased (घट्यो)
- Stayed about the same (उस्तै भयो)
- Increased (बढ्यो)

Have you ever given any of your PrEP pills to another person?
के तपाईले कहिलै आफ्नो PrEP पिल्स अरुलाई दिनु भएको छ?

- No (छैन)
- Yes (छ)

Do you know anyone else who uses PrEP?
के तपाईले PrEP प्रयोग गर्ने व्यक्तिलाई चिन्नु हुन्छ?

- No (छैन)
- Yes (छ)

Would you be willing to use or continue using PrEP to reduce your risk of getting HIV?
एचआईभीको जोखिम कम गर्न लाई के तपाईले भविष्यमा वा अहिले प्रयोग गर्दै आएको प्रेप को प्रयोग निरन्तर दिनु हुन्छ?

- No (गर्दिन)
- Yes (गर्छु)

Before taking this survey, have you ever heard about the injectable version of PrEP?
यो अध्यनमा भाग लिनु अघि के तपाईलाई प्रेप सुई देखि नि लिन सकिन्छ भन्ने थाहा थियो?

- No (थिएन)
- Yes (थियो)

Injectable PrEP involves receiving an injection of antiretroviral medication (called Cabotegravir). It involves an injection in your butt that is received every two months at a clinic. It has been found to be as safe and effective as taking daily oral PrEP.
सुई देखि दिने प्रेप भनेको एक antiretroviral औषधि हो जसलाई लाई काबोटेग्रविर पनि भनिन्छ| यो औषधि हरेक दुई दुई महिना सुई देखि पछाडि बटमा लगाउने गरिन्छ| यो औषधि पनि खाने प्रेप जत्तिकै प्रभाबकारी र सुरक्षित छ|

If an injectable PrEP were to be available in Nepal, would you be willing to take it?

सुई देखि लिने प्रेप नेपालमा उपलब्ध भए के तपाईले प्रयोग गर्नु हुन्थ्यो?

- No (गर्ने थिएन)
- Yes (थिए)

What is the maximum price you'd be willing to pay for a single dose of injectable PrEP?
तपाईले सुई देखि लिने एक डोज प्रेपको अधिकतम् कति सम्म तिर्न चाहनुहुन्छ?

- NRs 501-1,000 (नेरु ५००१- १०००)
- NRs 1,001-1,500 (नेरु १००१ -१५००)
- NRs 1,501-2,000 (नेरु १५०१ - २०००)
- NRs 2,001-2,500 (नेरु २००१ - २५००)
- More than NRs 2,500 (२५०० भन्दा धेरै)

If Nepal provided all types of PrEP at no cost (i.e., free), which type of PrEP would you most prefer to take
यदि सबै प्रकारका प्रेपहरु सर्बसुलभ उपलब्ध भए तपाईले कुन प्रयोग गर्न चाहनुहुन्छ?

- Daily oral PrEP (involves taking one pill a day) {दैनिक मुख देखि खाने प्रेप (दिनमा एक चक्की)}
- On-demand PrEP (involves taking two pills before sex one pill daily for two days after) {आवश्यकता अनुसार (दुई मात्रा खाने पिल्स, एक एक पिल्स यौन सम्पर्क भएको दुई दिन पछि एक एक मात्रा दिनमा)}
- Injectable PrEP (involves injection in your butt that you receive every two months at a clinic) {सुई देखि लिने प्रेप (दुई दुई महिनाको अन्तरहालमा स्वास्थ्य संस्था देखि पछाडि बटमा लगाउने)}
- None (कुनै पनि प्रयोग गर्दिन)

PEP

Before participating in this survey, have you ever heard of PEP, Post-Exposure Prophylaxis?
के तपाईले सम्पर्क पछिको प्रोफिल्याक्लिसस बारेमा सुन्नु भएको छ ?

- No (छैन)
- Yes (छ)

Where or how did you hear about PEP? Select all that apply.
कहाँ र कसरी सुन्नु भएको हो ? (तल मिल्ने जति सबै छान्नुहोस्)

- ☐ Printed Media (i.e., Newspaper, magazine) (पत्र पत्रिका)
- ☐ Online (अनलाइन)
- ☐ Social networking app (e.g., Facebook, Grindr, Hornet) (सामाजिक संजाल जस्तै फेसबोक, ग्रिंदर, हर्नेट)
- ☐ Medical Provider (स्वास्थ्य सेवा प्रादायकहरु संग)
- ☐ NGO's एन जी ओ
- ☐ HIV-related clinical research एचआईभी संबन्धित क्लिनिकल अनुसन्धान
- ☐ [ ] Other (specify): अन्य भए उल्लेख गर्नु होस्

Do you know anyone who has used PEP?
के तपाईले सम्पर्क पछिको प्रोफिल्याक्लिसस प्रयोग गर्ने मान्छे चिन्नु भएको छ ?

- ○ No (छैन)
- ○ Yes (छ)
- ○ Don't know (थाहा छैन)

PEP (post-exposure prophylaxis) means taking medicine to prevent HIV after a possible exposure. PEP should be used only in emergency situations and must be started within 72 hours after recent possible exposure to HIV.
सम्पर्क पछिको प्रोफिल्याक्लिसस भनेको एचआईभी भएको मान्छेसंगको सम्पर्क पछि एचआईभीको रोगथामको लागि खाने औषधि हो। यो सम्पर्कमा आएको ७२ घण्टा अघि नै आकस्मित बेलामा मात्र सेवन गर्नु पर्छ।

Have you ever used PEP?
के तपाईले सम्पर्क पछिको प्रोफिल्याक्लिसस प्रयोग गर्नु भएको छ?

- ○ No (छैन)
- ○ Yes (छ)

During the last time you used PEP, where did you get it?
अन्तिम पटक सम्पर्क पछिको प्रोफिल्याक्लिसस प्रयोग गर्दा कहाँ देखि लिनु भएको थियो?

- Government clinic/hospital (सरकारी अस्पताल)
- Private clinic/hospital (निजि अस्पताल)
- Community-based clinic (सामुदायिक स्वास्थ्य चौकी)
- Online (अनलाईन)
- [ ] Other (specify): (अन्य भए उल्लेख गर्नुहोस्)

During the last time you used PEP, did you miss any dose?
के तपाईले अन्तिम पटक सम्पर्क पछिको प्रोफिल्याक्लिसस प्रयोग गर्दा कुनै मात्रा छुटाउनु भएको थियो?

- No (छैन)
- Yes (छ)
- Unsure (थाहा छैन)

Throughout your lifetime, how many times have you used PEP?
तपाईले जीबनमा कति पटक सम्पर्क पछिको प्रोफिल्याक्लिसस प्रयोग गर्नु भएको छ?

- Never (कहिले पनि गरेको छैन)
- Once (एक पटक)
- Twice (दुई पटक)
- More than twice (दुई पटक भन्दा धेरै)

Do you know where or how to get PEP if you needed it?
के तपाईलाई सम्पर्क पछिको प्रोफिल्याक्लिसस कहाँ र कसरी पाउने भन्ने जानकारी छ?

- No (छैन)
- Yes (छ)

If you had potential exposure to HIV, would you be willing to use PEP?
यदि तपाई एचआईभी संक्रमणको सम्भाबित जोखिममा भए सम्पर्क पछिको प्रोफिल्याक्लिसस प्रयोग गर्नु हुन्छ?

- No (छैन)
- Yes (छ)

Healthcare & Services Utilization

This next section will include questions about health care and service utilization.
यस खण्डमा स्वास्थ्य सेवा र सेवा को उपयोग को बारे मा प्रश्नहरू सोधिने छ ।

Do you have any kind of health care coverage (including health insurance)?
के तपाईंसँग कुनै प्रकारको स्वास्थ्य हेरचाह गर्ने सुबिधा छ (स्वास्थ्य बीमा सहित)?

- No (छैन)
- Yes (छ)

A person’s appearance, style, or dress may affect the way people think of them. On average, how do you think people would describe your appearance, style, or dress?
कुनै पनि व्यक्तिको रुप शैली वा बर्णनले सोचाईमा असर पर्न सक्छ। तपाईलाई मानिसहरुले तपाईको रुप शैली वा पहिरनलाई कसरी बर्णन गर्छन् जस्तो लाग्छ ?

- Very Feminine (एकदमै महिलाको जस्तो)
- Mostly Feminine (धेरैजसो महिलाको जस्तो)
- Somewhat Feminine (केहि हदसम्म महिलाको जस्तो)
- Equally Feminine and Masculine (समानरुपमा महिला र पुरुषको जस्तो)
- Somewhat Masculine (केहि हदसम्म पुरुषको जस्तो)
- Mostly Masculine (धेरैजसो पुरुषको जस्तो)
- Very Masculine (एकदमै पुरुषको जस्तो)

A person’s mannerisms (such as the way they walk or talk) may affect the way people think of them. On average, how do you think people would describe your mannerisms?
कुनै व्यक्तिको चालचलनले (जस्तै: तिनीहरूको हिँडडुल गर्ने वा बोल्ने तरिका) मानिसहरूले तिनीहरूबारे सोच्ने तरिकामा असर पार्न सक्छ। तपाईलाई मानिसहरुले तपाईको चालचलनलाई कसरी बर्णन गर्छन् जस्तो लाग्छ ?

- Very Feminine (एकदमै महिलाको जस्तो)
- Mostly Feminine (धेरैजसो महिलाको जस्तो)
- Somewhat Feminine (केहि हदसम्म महिलाको जस्तो)
- Equally Feminine and Masculine (समानरुपमा महिला र पुरुषको जस्तो)
- Somewhat Masculine (केहि हदसम्म पुरुषको जस्तो)
- Mostly Masculine (धेरैजसो पुरुषको जस्तो)

- Very Masculine (एकदमै पुरुषको जस्तो)

Do you have any one person you think of as your personal doctor or healthcare provider?
के तपाईंको आफ्नो व्यक्तिगत डाक्टर वा स्वास्थ्यकर्मीको रूपमा सोच्ने कुनै व्यक्ति हुनुहुन्छ?

- No, no particular person (छैन)
- Yes, only one person (छ एक जना मात्र)
- Yes, more than one person (छ, एक भन्दा धेरै)

Please rate your level of agreement: When seeking healthcare, do you worry about being negatively judged because of your gender identity or sexual orientation?
के तपाईको लैगिंक पहिचानको कारणले तपाईलाई नकारात्मकरुपले लिन्छन भनेर स्वास्थ्य सेवा लिन जादा चिन्तित हुनु हुन्छ? (कृपया तपाईंको सहमतिको स्तर रेट गर्नुहोस्)

- Strongly agree (पूर्णरुपमा सहमत)
- Agree (सहमत)
- Neither agree nor disagree (न सहमत न असहमत)
- Disagree (असहमत)
- Strongly disagree (पूर्णरुमा असहमत)

Please rate your level of agreement: When seeking healthcare, do you worry that diagnoses of your health may be negatively affected by your gender identity or sexual orientation?
के तपाईको स्वास्थ्य निदानलाई लैगिंक पहिचानले नराम्रो तरिकाले असर गर्छ भनेर चिन्तित हुनु हुन्छ ? (कृपया तपाईंको सहमतिको स्तर रेट गर्नुहोस्)

- Strongly agree (पूर्ण रुपले सहमत)
- Agree (सहमत)
- Neither agree nor disagree (न सहमत न असहमत)
- Disagree (असहमत)
- Strongly disagree (पूर्ण रुपले असहमत)

When was the last time you visited your doctor or healthcare provider?

तपाई अन्तिम चोटी डाक्टर वा स्वास्थ्यकर्मीसंग गएको कहिले हो ?

- ○ Within the past 6 months (बिगत ६ महिना भित्र)
- ○ Within the past year (12 months) (बिगत १२ महिना भित्र)
- ○ Within the past 2 years (बिगत २ बर्ष भित्र)
- ○ 2 years or more (बिगत दुई बर्ष वा त्यो भन्दा धेरै)
- ○ Never (कहिलै पनि गएको छैन)

Of the following reasons, what cause you to not seek medical care within the past 12 months? Check all that apply.
बिगतको १२ महिनामा तपाई स्वास्थ्य सेवा लिन नजानुको कारण के हो? (मिल्ने सबै छान्नुहोस्)

- ☐ I have no health insurance (स्वास्थ्य बिमा नभएर)
- ☐ Cost (पैसा नभएर)
- ☐ Lack of transportation (यातायात नभएर)
- ☐ Fear of discrimination (भेदभावको डर)
- ☐ Lack of available appointments (पालो नपाएर)
- ☐ Clinic is too far (धेरै टाडा भएर)
- ☐ I did not need medical care (स्वास्थ्य सेवा नचाहिएर)
- ☐ It was not covered by my health insurance (स्वास्थ्य बिमाले बेहोर्न नसकेर)
- ☐ ________ Other (specify): (अन्य भए खुलाउनुहोस्)

I doubt that my health care providers really care about me as a person.
मलाई स्वास्थ्यसेवा प्रदायकहरुले एकरुपले हेर्नु हुन्छ भन्ने कुरामा शङ्का छ ?

- ○ Strongly disagree (पूर्ण असहमत)
- ○ Disagree (असहमत)
- ○ Neither Agree nor Disagree (न सहमत न असहमत)
- ○ Agree (सहमत)
- ○ Strongly Agree (पूर्ण सहमत)

My health care providers are usually considerate of my needs and puts them first.
स्वास्थ्य सेवा प्रदायकहरूले मेरो आवश्यकताहरुलाई विचार गरेर प्राथमिकतामा राख्नु हुन्छ ?

- Strongly disagree (पूर्ण असहमत)
- Disagree (असहमत)
- Neither Agree nor Disagree (न सहमत न असहमत)
- Agree (सहमत)
- Strongly Agree (पूर्ण सहमत)

I trust my health care providers so much I always try to follow their advice.
मैले मेरा स्वास्थ्य सेवा प्रदायकहरुलाई पूर्ण बिश्वास गर्छु र उनीहरुको सुझाबलाई पालन गर्ने प्रयास गर्छु ?

- Strongly disagree (पूर्ण असहमत)
- Disagree (असहमत)
- Neither Agree nor Disagree (न सहमत न असहमत)
- Agree (सहमत)
- Strongly Agree (पूर्ण सहमत)

If my health care providers tell me something is so, then it must be true.
यदि मलाई स्वास्थ्यकर्मीले केहि भन्नु हुन्छ भने त्यो पूर्ण रुपले सत्य वा ठिक हुनु पर्छ

- Strongly Disagree (पूर्ण असहमत)
- Disagree (असहमत)
- Neither Agree nor Disagree (न सहमत न असहमत)
- Agree (सहमत)
- Strongly Agree (पूर्ण सहमत)

I sometimes distrust my health care providers' opinion and would like a second one.
म कहिलेकाहीं मेरा स्वास्थ्य सेवा प्रदायकहरूको विचारमा विश्वास गर्दिन र अरुको विचार मन पराउछु

- Strongly Disagree (पूर्ण असहमत)
- Disagree (असहमत)
- Neither Agree nor Disagree (न सहमत न असहमत)
- Agree (सहमत)
- Strongly Agree (पूर्ण सहमत)

I trust my health care providers' judgments about my medical care.
मेरो स्वास्थ्य हेरचाहाको लागि मैले स्वास्थ्य सेवा प्रदायकहरूको निर्णयमा विश्वास गर्दछु।

- Strongly Disagree (पूर्ण असहमत)
- Disagree (असहमत)
- Neither Agree nor Disagree (न सहमत न असहमत)
- Agree (सहमत)
- Strongly Agree (पूर्ण सहमत)

I feel my health care providers do not do everything they should for my medical care.
मलाई मेरा स्वास्थ्य हेरचाह प्रदायकहरूले मेरो चिकित्सा हेरचाहको लागि गर्नु पर्ने सबै कुरा गर्दैनन् जस्तो महसुस गर्छु।

- Strongly Disagree (पूर्ण असहमत)
- Disagree (असहमत)
- Neither Agree nor Disagree (न सहमत न असहमत)
- Agree (सहमत)
- Strongly Agree (पूर्ण सहमत)

I trust my health care providers to put my medical needs above all other considerations when treating my medical problems.
मेरा स्वास्थ्य सेवा प्रदायकहरूले मेरो चिकित्सा समस्याहरूको उपचार गर्दा मेरो समस्यालाई प्राथमिकतामा राखेर उपचार गर्छन भन्ने विश्वास गर्छु।

- Strongly Disagree (पूर्ण असहमत)
- Disagree (असहमत)
- Neither Agree nor Disagree (न सहमत न असहमत)
- Agree (सहमत)
- Strongly Agree (पूर्ण सहमत)

My health care providers are real experts in taking care of medical problems.
मलाई मेरा स्वास्थ्य सेवा प्रदायकहरू चिकित्सा समस्याहरूको हेरचाह गर्ने वास्तविक विशेषज्ञहरू हुन् लाग्छ।

- Strongly Disagree (पूर्ण असहमत)

- Disagree (असहमत)
- Neither Agree nor Disagree (न सहमत न असहमत)
- Agree (सहमत)
- Strongly Agree (पूर्ण सहमत)

I sometimes worry that my health care providers may not keep the information we discuss totally private.
मलाई कतिबेला स्वास्थ्य सेवा प्रदायकहरु संग व्यक्तिगतरुपमा भएका छलफलका जानकारीहरु गुप्य हुदैनन् भनेर चिन्ता हुन्छ।

- Strongly Disagree (पूर्ण असहमत)
- Disagree (असहमत)
- Neither Agree nor Disagree (न सहमत न असहमत)
- Agree (सहमत)
- Strongly Agree (पूर्ण सहमत)

eHealth/mHealth ई हेल्थ/एम हेल्थ

We are just going to ask a few questions about technology and your use of smartphones and internet.
हामी तपाईलाई यस खण्डमा टेक्नोलोजी, स्मार्टफोन र इन्टरनेटको प्रयोगको बारेमा केही प्रश्नहरू सोध्ने छौ

Do you own or have access to the following devices on a daily basis? Check all that apply.
के तपाईं दैनिक रूपमा निम्न उपकरणहरूको पहुँचमा हुनु हुन्छ? मिल्ने सबैमा चिन्ह लगाउनुहोस् ।

- ☐ Landline telephone (ल्यान्डलाइन टेलिफोन)
- ☐ Mobile phone (without Internet access) {मोबाइल फोन (इन्टरनेट नभएको)}
- ☐ Mobile phone (with Internet access, i.e., a smartphone) (इन्टरनेट भएको मोबाइल फोन)
- ☐ Tablet (e.g., iPad, Samsung, Galaxy Tab, Kindle, etc.) {ट्याब्लेट (जस्तै, आईप्याड, सैमसंग, ग्यालेक्सी ट्याब, किन्डल, आदि)}
- ☐ Laptop (ल्यापटप)
- ☐ Personal Computer (PC) (कम्प्युटर)
- ☐ I do not have access to any of the previously mentioned devices on a daily basis (म माथि उल्लेख कुनै पनि उपकरणको दैनिक पहुचमा छैन)

How often do you use the following?
तपाईं निम्न उपकरणहरु कति पटक प्रयोग गर्नुहुन्छ?

	Never (कहिले पनि छैन)	Rarely (विरलै)	Sometimes (कहिले कहिँ)	Often (प्राय)	All the time (सधैँ)
Landline (ल्यान्डलाइन टेलिफोन	○	○	○	○	○
Mobile Phone (without Internet access) (इन्टरनेट नभएको मोबाइल फोन)	○	○	○	○	○
Mobile Phone (with Internet access - smartphone) (इन्टरनेट भएको मोबाइल फोन)	○	○	○	○	○
Tablet (e.g., iPad, Samsung, Galaxy Tab, Kindle) {ट्याब्लेट (जस्तै, आईप्याड, सैमसंग, ग्यालेक्सी ट्याब, किन्डल, आदि)}	○	○	○	○	○
Laptop (ल्यापटप)	○	○	○	○	○
Personal Computer (PC) (कम्प्युटर)	○	○	○	○	○

Please indicate how often you engage in the following activities on your mobile phone or Smartphone:
तपाईं आफ्नो मोबाइल फोन वा स्मार्टफोन मा निम्न गतिविधिहरुमा कति संलग्न हुनु हुन्छ :

	Never (कहिल्यै पनि गर्दिन)	Rarely (विरलै)	Sometimes (कहिलेकाहीँ)	Often (प्राय)	All the time (सधै)
Make or receive phone calls (फोन गर्ने वा उठाउने)	○	○	○	○	○
Send or receive text messages (सन्देश पठाउन वा प्राप्त गर्न)	○	○	○	○	○

	Never (कहिल्यै पनि गर्दिन)	Rarely (विरलै)	Sometimes (कहिलेकाहीँ)	Often (प्राय)	All the time (सधै)
Browse gay social media sites and applications (e.g., Grindr, Jack'd, Plantet Romeo) {समलिङ्गी सामाजिक मिडिया वा साइटहरू (ग्रिंद्र, ज्याक, प्लानेट, रोमियो)}	○	○	○	○	○
Online social networking (e.g., Facebook, Twitter, Instagram, Snapchat, MySpace) {सामाजिक संजाल (फेसबुक, ट्वीटर, इन्स्टाग्राम,स्नाप च्याट, ई स्पेस)}	○	○	○	○	○
Use health-related apps (स्वास्थ्य सम्बन्धि एपहरू)	○	○	○	○	○
Search for health-related information (स्वास्थ्य सम्बन्धी जानकारी खोज्नुहोस्)	○	○	○	○	○
Send or receive emails (ईमेल पठाउन वा प्राप्त गर्न)	○	○	○	○	○
Listen to music (गीत सुन्न)	○	○	○	○	○
Take a picture (फोटो लिन)	○	○	○	○	○
Record a video (भिडियो खिच्न)	○	○	○	○	○
Watch videos (भिडियो हेर्न)	○	○	○	○	○
Download or watch pornographic video/picture (पोर्नोग्राफिक भिडियो/ तस्विर डाउनलोड गर्न वा हेर्न)	○	○	○	○	○
Having phone sex (फोन सेक्स गर्न)	○	○	○	○	○

	Never (कहिल्यै पनि गर्दिन)	Rarely (विरलै)	Sometimes (कहिलेकाहीँ)	Often (प्राय)	All the time (सधै)
Having video sex (cam sex) (भिडीयो सेक्स गर्न)	○	○	○	○	○
Play games (ग्याम खेल्न)	○	○	○	○	○

Do you currently have DAILY access to the Internet?
के तपाइँसँग हाल इन्टरनेटमा दैनिक पहुँच छ?

- ○ No (छैन)
- ○ Yes (छ)

On average, how many hours do you spend daily to access the Internet? Please enter just the number.
औसतमा दिनमा तपाईं कति घण्टा इन्टरनेट चलाउनु हुन्छ? कृपया नम्बरमा मात्र लेख्नुहोस्।

[ ]

Which is your PRIMARY device for accessing the Internet? Note: you may use multiple devices to access the Internet, please select the one you use the most.
इन्टरनेटको लागि तपाईंको प्राथमिक उपकरण कुन हो ? (तल मध्ये इन्टरनेटको लागि तपाईले सबै भन्दा धेरै प्रयोग गर्ने उपकरण छानुहोस्)

- ○ Smartphone (स्मार्ट फोन)
- ○ Tablet (ट्याब्लेट)
- ○ Laptop (ल्यापटप)
- ○ Personal Computer (कम्प्युटर)
- ○ [ ] Other (अन्य)

How willing are you to use mobile technologies such as mobile phones, tablets, or other similar devices to:
तपाईं मोबाइल फोन, ट्याब्लेट वा अन्य त्यस्तै प्रविधिहरू तल दिएको कामको लागि प्रयोग गर्न कत्तिको इच्छुक हुनुहुन्छ:

	Never (कहिल्यै पनि चाहन्न)	Rarely (विरलै)	Sometimes (कहिलेकाहीं)	Often (प्राय)	All the time (सधै)
Receive reminders to take your medication? (तपाईं औषधि लिनका लागि रिमाइन्डरहरू प्रयोग गर्न कत्तिको इच्छुक हुनुहुन्छ?)	○	○	○	○	○
Monitor your cravings for chem? (तपाईले केम को इच्छाको अनुगमन गर्न कत्तिको इच्छुक हुनुहुन्छ ?)	○	○	○	○	○
Track your drug use history as a tool to stop using drugs? (तपाई लागूपदार्थ सेवन रोक्न कुनै उपकरणको प्रयोग गर्न कत्तिको इच्छुक हुनुहुन्छ?)	○	○	○	○	○
Record sexual history to reduce risky sexual behaviors? (तपाई जोखिमपूर्ण यौन व्यवहार कम गर्न यौन इतिहास रेकर्ड गर्न कत्तिको इच्छुक हुनुहुन्छ?)	○	○	○	○	○
Receive information about drug use treatment? (तपाई लागूऔषध सेवन उपचारको बारेमा जानकारी प्राप्त गर्न कत्तिको इच्छुक हुनुहुन्छ?)	○	○	○	○	○
Receive information on HIV prevention? (तपाई एचआईभी रोगथामको बारेमा जानकारी प्राप्त गर्न कत्तिको चाहनुहुन्छ?)	○	○	○	○	○
e-consult with doctors (e.g., Telehealth)? तपाईले चिकित्सक संग टेलीफोन वा मोबाइल फोन प्रयोग गरेर परामर्श लिन कत्तिको चाहनुहुन्छ ?	○	○	○	○	○
Order HIV prevention supplies (e.g., condoms, lube, PrEP/PEP medications)? (तपाईले प्रविधिहरूको प्रयोग गरेर जस्तै मोबाइल इन्टरनेट एचआईभी रोगथामका सामनहरु जस्तै काण्डम, लुबहरु मगाउन कत्तिको चाहनुहुन्छ?)	○	○	○	○	○
Receive information on mental health services? (तपाईले मानसिक स्वास्थ्य सेवाहरुको बारेमा जानकारी लिन कत्तिको चाहनुहुन्छ?)	○	○	○	○	○

	Never (कहिल्यै पनि चाहन्न)	Rarely (विरलै)	Sometimes (कहिलेकाहीं)	Often (प्राय)	All the time (सधैं)
Virtual support group for mental health? तपाईले मानसिक स्वास्थ्यको लागि इन्टरनेटमा भएका केहि समहू देखि सहयोग लिन कत्तिको लिन चाहनुहुन्छ ?	○	○	○	○	○

Which of the following ways would you prefer the most to: (choose one)
निम्न काम गर्नको लागि तल मध्य कुन तरिका तपाईंलाई सबैभन्दा बढी मन पर्छ: (एउटा रोज्नुहोस्)

	Phone Call (फोन कल)	Text Messages (सन्देश पठाउन वा प्राप्त गर्न)	Apps (एप्स)	Websites (वेबसाइट)	N/A (लागु हुँदैन)
Receive reminders to take your medication? (औषधि सेवनको लागि रिमाइन्डरहरू प्रयोग)	○	○	○	○	○
Monitor your cravings for chem? (केम को इच्छाको अनुगमन)	○	○	○	○	○
Track your drug use history as a tool to stop using drugs? (लागूपदार्थ सेवन रोक्न कुनै उपकरणको प्रयोग गरेर लागूऔषध प्रयोगको इतिहास हेर्न)	○	○	○	○	○
Record sexual history to reduce risky sexual behaviors? (जोखिमपूर्ण यौन व्यवहार कम गर्न यौन इतिहास रेकर्ड गर्न)	○	○	○	○	○
Receive information about drug use treatment? (लागूऔषध सेवन उपचारको बारेमा जानकारी प्राप्त गर्न)	○	○	○	○	○
Receive information on HIV prevention? (एचआईभी रोगथामको बारेमा जानकारी प्राप्त गर्न)	○	○	○	○	○

	Phone Call (फोन कल)	Text Messages (सन्देश पठाउन वा प्राप्त गर्न)	Apps (एप्स)	Websites (वेबसाइट)	N/A (लागु हुँदैन)
e-consult with doctors (e.g., Telehealth)? (चिकित्सक संग टेलीफोन वा मोबाइल फोन प्रयोग गरेर परामर्श लिन)	○	○	○	○	○
Order HIV prevention supplies (e.g., condoms, lube, PrEP/PEP medications)? (एचआईभी रोगथामका सामनहरु जस्तै काण्डम, लुबहरु मगाउन)	○	○	○	○	○
Receive information on mental health services? (मानसिक स्वास्थ्य सेवाहरुको बारेमा जानकारी लिन)	○	○	○	○	○
Virtual support group for mental health? (मानसिक स्वास्थ्यको लागि भर्चुअल रुपमा केहि समहू देखि सहयोग लिन)	○	○	○	○	○

How frequently are you willing to: तलका कामहरु गर्न तपाईं कत्तिको इच्छुक हुनुहुन्छ

	Never कहिले पनि हैन	Monthly मासिक	Weekly (साप्ताहिक)	Daily दैनिक
Receive reminders to take your medication? (औषधि सेवनको लागि रिमाइन्डरहरू प्रयोग)	○	○	○	○
Monitor your cravings for chem? (केम को इच्छाको अनुगमन)	○	○	○	○
Track your drug use history as a tool to stop using drugs? (लागूपदार्थ सेवन रोक्न कुनै उपकरणको प्रयोग गरेर लागूऔषध प्रयोगको इतिहास हेर्न)	○	○	○	○

	Never कहिले पनि हैन	Monthly मासिक	Weekly (साप्ताहिक)	Daily दैनिक
Record sexual history to reduce risky sexual behaviors? (जोखिमपूर्ण यौन व्यवहार कम गर्न यौन इतिहास रेकर्ड गर्न)	○	○	○	○
Receive information about drug use treatment? (लागूऔषध सेवन उपचारको बारेमा जानकारी प्राप्त गर्न)	○	○	○	○
Receive information on HIV prevention? (एचआईभी रोगथामको बारेमा जानकारी प्राप्त गर्न)	○	○	○	○
e-consult with doctors (e.g., Telehealth)? (चिकित्सक संग टेलीफोन वा मोबाइल फोन प्रयोग गरेर परामर्श लिन)	○	○	○	○
Order HIV prevention supplies (e.g., condoms, lube, PrEP/PEP medications)? (एचआईभी रोगथामका सामनहरु जस्तै काण्डम, लुबहरु मगाउन)	○	○	○	○
Receive information on mental health services? (मानसिक स्वास्थ्य सेवाहरुको बारेमा जानकारी लिन)	○	○	○	○

Which of the following LGBTQIA+ social media sites and applications do you currently use? Check all that apply.
तल मध्य तपाईले LGBTQIA को कुन सामाजिक संजाल वा याप अहिले प्रयोग गर्दै आउनु भएको छ ? (मिल्ने जति सबैमा टिक लगाउनुहोस्)

- ☐ Grindr (गरैण्डर)
- ☐ Blued (ब्लुएड)
- ☐ Jack'd (ज्याकड)

- ☐ Planet Romeo (प्लानेट रोमेओ)
- ☐ Manjam (मंजम)
- ☐ Dudesnude (डुडनुड)
- ☐ Micr (मिक्र)
- ☐ Scruff (स्क्रुफ्फ़)
- ☐ Growler (ग्रोव्लेर)
- ☐ Recon (रेकन)
- ☐ Hornet (हर्नेट)
- ☐ BarebackRT/BBRT (बरेब्याक)
- ☐ Manhunt (मनहन्ट)
- ☐ Other (अन्य)
- ☐ None (कुनै पनि हैन)

Stigma/Victimization Items लान्छना

This next section will ask you about your experiences with stigma and discrimination. Just a few more sections to go after this!
तपाईलाई यो खण्डमा भेदभावको अनुभवको बारेमा केहि प्रश्नहरु सोध्नेछौ । यस पछि अझै केहि खण्डहरू बाकी छन्!

Using the following answer options, please indicate how often you have experienced the following:
कृपया तल दिएका विकल्पहरू प्रयोग गरेर, तपाईंले निम्न अनुभव कति पटक गर्नुभएको छ भनेर भन्नु होस्:

	Never (कहिले पनि छैन)	Once or twice (एक वा दुई पटक)	A few times (केहि पटक)	Ma tim (पट
How often have you heard that men who have sex with men are not normal? (तपाईले पुरुषसँग यौनसम्पर्क गर्ने पुरुषहरू सामान्य हुँदैनन् भन्ने कुरा कति पटक सुन्नु भएको छ ?)	○	○	○	(
How often have you felt that your family was hurt or embarrassed because you have sex with men? (तपाईले पुरुषसँग यौनसम्पर्क गर्दा तपाईको परिवारलाई कहिले चिन्तित वा अपमानित भएको महसुस गर्नुभएको छ?)	○	○	○	(

	Never (कहिले पनि छैन)	Once or twice (एक वा दुई पटक)	A few times (केहि पटक)	Ma tim [illegible] पट
How often have you been made fun of or called names because you have sex with men? (तपाईले पुरुषसँग यौनसम्पर्क गरेको कारण कति पटक तपाईलाई जिस्काईको वा नराम्रो नाम ले बोलाईएको छ ?)	○	○	○	○
How often have you been hit or beaten up because you have sex with men? (पुरुष संग यौन सम्पर्क गरेको कारणले तपाईलाई कति पटक पिटिएको छ ?)	○	○	○	○
How often have you had to pretend that you do not have sex with men in order to be accepted? (तपाईलाई समाज वा साथीभाईले स्वीकारुन भनेर कति पटकसम्म पुरुषसँग यौनसम्पर्क नगरेको बहाना गर्नुपर्‍यो?)	○	○	○	○
How often has your family not accepted you because you have sex with men? (तपाईलाई कति पटक तपाईको परिवारले पुरुष संग यौन सम्पर्क गरेको कारणले स्वीकारेको छैन?)	○	○	○	○
How often have you lost your friends because you have sex with men? (पुरुषसँग यौनसम्पर्क गरेको कारणले कति पटक तपाईंले आफ्ना साथीहरू गुमाउनु भएको छ?)	○	○	○	○
How often have you been kicked out of school because you have sex with men? (पुरुषसँग यौनसम्पर्क गरेको कारणले तपाईलाई विद्यालय देखि कति पटक निकालिएको छ ?)	○	○	○	○
How often have you lost a place to live because you have sex with men? (पुरुषसँग यौनसम्पर्क गरेको कारण तपाईले आफु बस्दै आएको ठाउँ कतिपटक गुमाउनु परेको छ ?)	○	○	○	○
How often have you lost a job or career opportunity because you have sex with men? (पुरुषसँग यौनसम्पर्क गरेको कारण तपाईले कति पटक जागिर वा पेशागत अवसरहरु गुमाउनु परेको छ ?)	○	○	○	○
How often have you been verbally abused by police because you have sex with men? (पुरुषसँग यौनसम्पर्क गरेको कारणले कति पटक तपाईलाई प्रहरीले मौखिक दुर्व्यवहार गरेको छ?)	○	○	○	○
How often have you been physically abused by police because you have sex with men? (पुरुषसँग यौनसम्पर्क गरेको कारणले कति पटक तपाईलाई प्रहरीले शारीरिक दुर्व्यवहार गरेको छ?)	○	○	○	○
How often have you been sexually abused by police because you have sex with men? (पुरुषसँग यौनसम्पर्क गरेको कारणले कति पटक तपाईलाई प्रहरीले यौन दुर्व्यवहार गरेको छ?)	○	○	○	○
How often have you been blackmailed for money because you have sex with men? (तपाईलाई कहिलै पुरुषसंग यौन सम्पर्क गरेको कारण देखाएर फसाउने बाहानामा पैसा मागिएको छ ?)	○	○	○	○

Consider all incidents in which you were mistreated because of your sexual orientation. Select all that apply.
तपाईंको लैंगिक पहिचान कारणले तपाईं कस्कसको दुर्व्यवहारमा पर्नु भएको छ । मिल्ने जति सबैमा टिक लगाउनुहोस्

- ☐ From family (परिवार देखि)
- ☐ While in school (विद्यालयमा)
- ☐ From your employer or coworkers (सहकर्मीहरु देखि)
- ☐ From sex work clients (यौन ग्राहक देखि)
- ☐ From a medical provider (स्वास्थ्यकर्मी देखि)
- ☐ From a government official (सरकारी कर्मचारी देखि)
- ☐ While on the street or in public (सडकमा वा सार्बजनिक स्थलमा)
- ☐ From sexual partner(s) (यौन साथी देखि)
- ☐ From police (प्रहरी देखि)
- ☐ I was never mistreated because of my sexual orientation (म अहिले सम्म लैंगिक पहिचानको कारणले दुर्व्यवहारमा परेको छैन)
- ☐ ______ Other (specify): (अन्य भए उल्लेख गर्नु होस्)

How much were you bothered by this experience?
यो अनुभवले तपाईलाई कत्तिको दिक्क वा नराम्रो लाग्यो?

- ○ Not at all (केहि भएन)
- ○ A little (अलिकति)
- ○ Somewhat (केही हद सम्म)
- ○ A lot (धेरै)
- ○ Extremely (एकदम धेरै)

Have you ever been detained by the police, placed in lock-up, or imprisoned for any reason?
के तपाईलाई कुनै कारण बस् हिरासतमा वा थुनामा राखिएको छ ?

- ○ No (छैन)
- ○ Yes (छ)
- ○ Prefer not to answer (उल्लेख गर्न चाहन्न)

What were you detained, arrested, or imprisoned for? Select all that apply.
के कारणले गर्दा थुनामा वा हिरासतमा पर्नु भएको थियो ? (मिल्ने सबै मा टिक लगाउनुहोस्)

- ☐ Sex work (यौन कार्य)
- ☐ Drug use (लागूऔषधको सेवन)
- ☐ Selling drugs (लागूऔषध बेचबिखन)
- ☐ Theft (चोरी)
- ☐ Robbery (डकैती)
- ☐ Violence/assault (दुर्व्यवहार गरेर)
- ☐ ____________ Other (specify): (अन्य भए खुलाउनुहोस्)

Using the following answer options, please indicate how often a partner has done the following since you turned 18:
तपाईं 18 वर्षको भएदेखि जीबन साथी वा साथीले कति पटक निम्न कार्यहरू गर्नुभएको छ: (तलको मिल्ने उत्तर मा चिन्ह लगाउनुहोस्)

	Never (कहिले पनि छैन)	Rarely (विरलै)	Sometimes (कहिलेकाहीँ)	Fairly Often (प्रायः)	Frequently (बारम्बार)
Physically hurt you? (तपाईलाई कति पटक शारीरिक चोट जस्तै कुटपिट गर्नु भएको छ?)	○	○	○	○	○
Insulted or talked down to you? (तपाईलाई कतिपटक गाली वा होच्याउने गरेको छ ?)	○	○	○	○	○
Threatened you with harm? (तपाईलाई कति पटक धम्काउने थर्काउने गरेको छ ?)	○	○	○	○	○
Screamed or cursed at you? (तपाईलाई कति पटक चिच्याउने वा श्राप दिने काम गरेको छ ?)	○	○	○	○	○

	Never (कहिले पनि छैन)	Rarely (विरलै)	Sometimes (कहिलेकाहीँ)	Fairly Often (प्रायः)	Frequently (बारम्बार)
Forced you to do sexual acts that you were not comfortable with? (तपाईलाई कति पटक मन नपर्ने यौन कार्य गर्न बाध्य बनाएको छ ?)	○	○	○	○	○

Social Support

Now we are going to ask you about social support and some of the people close to you.
अब हामी तपाईलाई सामाजिक समर्थन र तपाईका नजिकका केही व्यक्तिहरूको बारेमा सोध्न जाँदैछौं।

Have you disclosed your sexual orientation to any of the following? Select all that apply.
के तपाईंले तल मध्ये कसैसंग आफ्नो यौन झुकाव खुलासा गर्नुभएको छ? लागू हुने सबै चयन गर्नुहोस्।

- ☐ Family members (परिवारको सदस्य संग)
- ☐ Friends (साथी)
- ☐ At workplace (काम गर्ने ठाउँमा)
- ☐ Healthcare provider (स्वास्थ्यकर्मी संग)
- ☐ No one (कसै संग पनि छैन)
- ☐ ______ Other (specify): (अन्य भए खुलाउनुहोस्)

What are the reasons that you have NOT disclosed that you are living and identifying full-time as MSM? Select all that apply.
तपाईले पुरुष संग यौन सम्पर्क गर्छु भनेर लैङ्गिक पहिचान नखुलाउनुको कारण के हो ? लागू हुने सबै चयन गर्नुहोस्।

- ☐ Family disapproves (परिवारले अस्वीकार गर्छ भन्ने डरले)
- ☐ Partner disapproves (पार्टनरले अस्वीकार गर्छ भन्ने डरले)
- ☐ Discrimination at work (काम गर्ने ठाउँमा भेदभाव हुने डरले)
- ☐ Discrimination at home (घरमा भेदभाव हुने डरले)
- ☐ Discrimination from healthcare provider (स्वास्थ्यकर्मीले भेदभाव गर्नु हुन्छ भन्ने डरले)
- ☐ Not out (केहि पनि छैन)
- ☐ ______ Other (specify): (अन्य भए खुलाउनुहोस्)

Does your family pressure you or force you to marry a female?
के तपाईलाई परिवार बाट केटि संग बिहे गर्न दबाब छ ?

- No (छैन)
- Yes (छ)

How supportive/accepting is the neighborhood in which you reside in towards sexual minority men?
तपाई बस्ने नजिकका छिमिकीहरु यौन अल्पसंख्यक पुरुषहरूप्रति कत्तिको सहयोगी र स्वीकार्ने हुनु हुन्छ ?

- Extremely supportive (अति सहयोगी)
- Mainly supportive (सहयोगी)
- Neutral (तटस्थ)
- Not supportive (असहयोगी)
- Extremely not supportive (अति असहयोगी)

How supportive is your family regarding your sexual orientation?
तपाईंको परिवार तपाईंको यौन झुकावमा प्रति कत्तिको सहयोगी छ?

- Mainly supportive (अति सहयोगी)
- Mainly supportive (सहयोगी)
- Neutral (तटस्थ)
- Not supportive (असहयोगी)
- Extremely not supportive (अति असहयोगी)

How supportive are your friends regarding your sexual orientation?
तपाईंका साथीहरु तपाईंको यौन झुकावमा प्रति कत्तिको सहयोगी छन्?

- Extremely supportive (अति सहयोगी)
- Mainly supportive (सहयोगी)
- Neutral (तटस्थ)
- Not supportive (असहयोगी)
- Extremely not supportive (अति असहयोगी)

How many other MSM do you know who also know you well and live in this same district? (Knowing someone is defined as being able to contact them and having had contact with them in the past 6 months). Please enter just the number.
तपाईले कति जना यै जिल्लाका वा ठाउँका पुरुष संग यौन सम्पर्क गर्ने पुरुषहरुलाई राम्रो संग चिन्नु भएको छ ? (कसैलाई चिन्नु भनेको उनीहरूलाई सम्पर्क गर्न सक्ने र उनीहरूसँग विगत ६ महिनामा सम्पर्क भएको रूपमा परिभाषित गरिएको छ)।

How many people are so close to you that you can count on them if you have great personal problems?
यदि तपाईंलाई ठूलो व्यक्तिगत समस्या आए कतिजना व्यक्तिहरु संग तपाईं पूर्ण भरोसा गर्न सक्नुहुन्छ ?

- None (कोहि पनि छैन)
- 1-2 (१ -२)
- 3-5 (३-५)
- 5+ (५ भन्दा धेरै)

How much interest and concern do people show in what you do?
तपाईंले गरेको काममा मानिसहरूले कत्तिको चासो र जिज्ञासा देखाउँछन्?

- None (कुनै पनि छैन)
- Little (थोरै)
- Uncertain (अनिश्चित)
- Some (केही)
- A lot (धेरै)

How easy is it to get practical help from relatives and neighbors if you should need it?
तपाईलाई आवश्यक परेमा आफन्त र छिमेकीबाट व्यावहारिक सहयोग पाउन कत्तिको सजिलो छ?

- Very difficult (धेरै गाह्रो)
- Difficult (गाह्रो)
- Possible (सम्भव छ)
- Easy (सजिलो)

○ Very easy (धेरै सजिलो)

Mental Health मानसिक स्वास्थ्य

We are at the second to last section! This section will ask some questions about how you're feeling mentally, and about your history with mental health.
हामी अन्त्य अन्त्यमा आईपुग्यौ | यस खण्डमा तपाईलाई मानसिक रूपमा कस्तो महसुस गरिरहनुभएको छ, र मानसिक स्वास्थ्यसँगको तपाईको अनुभव कस्तो छ बारेमा केही प्रश्नहरू सोध्छौ।

Over the last 2 weeks, how often have you been bothered by the following problems?
अव हामी तपाईलाई गएको २ हप्तामा एक दुई दिनमात्रै नभएर लगातार धेरै दिनसम्म मन दुःखी भईरहने, खिन्न लागिरहने वा नरमाईलो लागिरहने भएको थियो कि थिएन भन्ने वारेमा कुरा गर्छौं।

	Not at all (कत्ति पनि भएन)	Several days (कहिले काहीं भयो)	More than half the days (धेरै जसो भयो)	Nearly every day (सधै जसो भयो)
Little interest or pleasure in doing things (गएको २ हप्तामा तपाइलाई अन्य ब्यक्तिहरू जति रमाउँछन्, त्यत्तिको रमाउन नसक्ने खुसी हुन नसक्ने वा काम गर्न मन नलाग्ने कत्तिको भएको थियो ?)	○	○	○	○
Feeling down, depressed or hopeless (गएको २ हप्तामा तपाइलाई, दिक्क लाग्ने, निरास हुने वा केही गर्न सक्दिन जस्तो लाग्ने कत्तिको भएको थियो ?)	○	○	○	○
Trouble falling asleep, staying asleep, or sleeping too much (गएको २ हप्तामा तपाईंलाई राम्ररी सुत्न नसक्ने वा निन्द्रा मस्त नआउने वा अघिपछि भन्दा धेरै सुत्ने वा धेरै निन्द्रा लाग्ने कत्तिको भएको थियो ?)	○	○	○	○

	Not at all (कत्ति पनि भएन)	Several days (कहिले काहीं भयो)	More than half the days (धेरै जसो भयो)	Nearly every day (सधै जसो भयो)
Feeling tired or having little energy (गएको २ हप्तामा तपाईंलाई थकान महशुस हुने, शक्ति वा तागत् कम हुने कत्तिको महशुस भएको थियो ?)	○	○	○	○
Poor appetite or overeating (गएको २ हप्तामा तपाईंलाई खाना खान मन नलाग्ने वा रुचि नहुने वा अघिपछि भन्दा धेरै खान मन लाग्ने कत्तिको भएको थियो ?)	○	○	○	○
Feeling bad about yourself - or that you're a failure or have let yourself or your family down (गएको २ हप्तामा तपाई आफूले आफूलाई दोषी ठान्ने वा आफ्नो कारणले गर्दा आफू असफल भएको वा परिवार तल परेको कत्तिको महशुस गर्नु भएको थियो ? वा आफ्नो कारणले गर्दा समुदायमा आफ्नो तथा आफ्नो परिवारको ईज्जत गुमेको महशुस गर्ने ।)	○	○	○	○
Trouble concentrating on things, such as reading the newspaper or watching television (गएको २ हप्तामा तपाईंलाई ध्यान केन्द्रित गर्न वा एक चित्त हुन वा एक सुरले काम गर्न गाह्रो हुने कत्तिको भएको थियो ? (जस्तैः पत्रपत्रिका पढ्न वा टी.भी. हेर्न, तरकारी पकाउन, ध्यान दिएर अन्य कुनैपनि काम गर्न)	○	○	○	○

	Not at all (कत्ति पनि भएन)	Several days (कहिले काहीं भयो)	More than half the days (धेरै जसो भयो)	Nearly every day (सधै जसो भयो)
Moving or speaking so slowly that other people could have noticed. Or the opposite - being so fidgety or restless that you have been moving around a lot more than usual (गएको २ हप्तामा अरू ब्यक्तिले पनि याद गर्ने गरी तपाई बिस्तारै बोल्ने वा हिड्ने गर्नुभएको अथवा छट्पट्टी भएर चाहिने भन्दा बढी हिंडडुल गर्ने कत्तिको गर्नुभएको थियो ?)	○	○	○	○
Thoughts that you would be better off dead or hurting yourself in some way (गएको २ हप्तामा तपाईंलाई मरौं मरौं जस्तो लाग्ने, आफ्नो ज्यान आफैं लिने वा आत्महत्या गर्ने खालका विचारहरू कत्तिको आयो ? (जस्तैः हात काट्ने, विष सेवन गर्ने, हाम फाल्ने, भित्तामा टाउको ठोक्काउने आदि)	○	○	○	○

Have you ever in your life had thoughts of killing yourself?
के तपाईलाई जीबनमा आत्महत्या गर्ने सोच आएको थियो?

- ○ No (आएको छैन)
- ○ Yes (आएको छ)

How often did you have any thoughts about ending your own life in the past 12 months?
विगत १२ महिनामा तपाईलाई आफ्नै जीवन समाप्त गर्ने बारे कति पटक सोच्नु भएको थियो?

- ○ Many times (धेरै पटक)
- ○ A few times (केही पटक)
- ○ Once or twice (एक वा दुई पटक)

- Never (कहिले पनि छैन)

Have you ever made a plan to commit suicide?
के तपाईले जीबनमा आत्माहत्या गर्ने योजना गराउनु भएको थियो?

- No (गराएको छैन)
- Yes (गराएको छु)

Have you ever attempted suicide?
के तपाईले जीबनमा आत्माहत्या गर्ने प्रयास गर्नु भएको छ?

- No (छैन)
- Yes (छ)
- Yes, more than once (एक पटक भन्दा धेरै गरेको छु)

Have you seen a mental health professional in the last 12 months?
बिगतको १२ महिनामा के तपाईले मानसिक स्वास्थ्य स्वास्थ्यकर्मी संग परामर्श लिनु भएको छ?

- No, but I didn't need to (लिएको छैन र आवश्यक पनि छैन)
- No, and I needed to (छैन तर मलाई आवश्यक छ)
- Yes (छ)

Why didn't you see a mental health care professional in the past 12 months? Select all that apply.
तपाईंले विगत १२ महिनामा मानसिक स्वास्थ्य परामर्श नलिनुका कारण के के हुन्? तल मिल्ने सबै उत्तरमा छान्नुहोस्।

- ☐ Didn't need mental health services (मानसिक स्वास्थ्य परामर्श नचाहिएर)
- ☐ Didn't know where to go (कहाँ जाने थाहा नभएर)
- ☐ Afraid they will discriminate against me (भेदभाव गर्ने डरले)
- ☐ Afraid they will tell others about my gender identity (लैङ्गिक पहिचान खुलाउने सक्ने डरले)
- ☐ Can't afford it (मेरो पहुचमा नभएर)
- ☐ ________ Other (specify): (अन्य भए खुलाउनुहोस्)

In the past year, have you had any problems getting mental health services because of your gender identity?
बिगतको बर्षमा के तपाईलाई लैङ्गिक पहिचान को कारण मानसिक स्वास्थ्य सेवाहरु प्राप्त गर्न कुनै समस्या भएको थियो?

- ○ No, but I didn't seek the services (मैले कुनै पनि सेवाहरु लिएको छैन र भेदभाव पनि महसुस भएको छैन)
- ○ No and I did seek these services (सेवा लिएको छु तर भेदभाव महसुस गरेको छैन)
- ○ Yes (थियो)

Did the mental health services you received meet your MSM-specific mental health care needs?
के तपाईंले प्राप्त गर्नुभएको मानसिक स्वास्थ्य सेवाहरूले बिषेश गरि MSM (पुरुषसँग यौन सम्पर्क गर्ने पुरुषहरूको) मानसिक स्वास्थ्य हेरचाह आवश्यकताहरू पूरा गर्यो?

- ○ No (गरेन)
- ○ Yes (गर्यो)
- ○ I did not receive mental health services (मैले मानसिक स्वास्थ्य सेवा नै लिएको छैन)

Have you seen a medical provider in the past 12 months?
बिगतको १२ महिनामा के तपाईले मानसिक चिकित्सक संग परामर्श लिनु भएको छ?

- ○ No (छैन)
- ○ Yes (छु)

Why didn't you see a medical provider in the past 12 months? Select all that apply.
तपाईंले विगत १२ महिनामा मानसिक चिकित्सक संग परामर्श नलिनुका कारण के के हुन्? तल मिल्ने सबै उत्तरमा छान्नुहोस्।

- ☐ Didn't need mental health services (मानसिक स्वास्थ्य परामर्श नचाहिएर)
- ☐ Didn't know where to go (कहाँ जाने थाहा नभएर)
- ☐ Afraid they will discriminate against me (म विरुद्ध भेदभाव गर्ने डरले)
- ☐ Afraid they will tell others about my gender identity (मेरो लैङ्गिक पहिचानको बारेमा अरूलाई भन्लान भन्ने डरले)
- ☐ Can't afford it (खर्च नभएर)

☐ [ ] Other (specify) अन्य भए खुलाउनुहोस्

In the past year, have you had any problems getting a medical provider because of your gender identity?
बिगतको बर्षमा के तपाईलाई लैङ्गिक पहिचान को कारण मानसिक चिकित्सक प्राप्त गर्न कुनै समस्या भएको थियो?

- No, but I didn't see these services (मैले कुनै पनि सेवाहरु लिएको छैन र भेदभाव पनि महसुस भएको छैन)
- No and I did seek these services (सेवा लिएको छु तर भेदभाव महसुस गरेको छैन)
- Yes (थियो)

Sleep

How likely are you to doze off or fall asleep in the following situations? You should rate your chances of dozing off, not just feeling tired. Even if you have not done some of these things recently try to determine how they would have affected you. For each situation, decide whether or not you would have: · No chance of dozing =0 · Slight chance of dozing =1 · Moderate chance of dozing =2 · High chance of dozing =3
तपाईं निम्न अवस्थाहरूमा निदाउने वा सुत्ने सम्भावना कत्तिको छ? तपाईंले थकित महसुस मात्र होइन, सुत्ने सम्भावना मूल्याङ्कन गर्नुपर्छ। तपाईंले हालसालै यी केही कुराहरू गर्नुभएको छैन भने पनि तिनीहरूले तपाईंलाई कस्तो असर पार्छ भनेर निर्धारण गर्ने प्रयास गर्नुहोस्। प्रत्येक अवस्थाको लागि, तपाईंसँग हुन्छ वा छैन भन्ने निर्णय गर्नुहोस्: · निदाउने सम्भावना छैन = ० · निद्रा लाग्ने अलिकति सम्भावना छ = १ · निद्रा लाग्ने मध्यम सम्भावना = २ · निदाउने उच्च सम्भावना = ३

	No chance of dozing (निदाउन सम्भावना छैन)	Slight chance of dozing (निद्रा लाग्ने अलिकति सम्भावना छ)	Moderate chance of dozing (निद्रा लाग्ने मध्यम सम्भावना छ)	High chance of dozing (निदाउने उच्च सम्भावना छ)
Sitting and reading (बस्दै गर्दा वा पढ्दै गर्दा)	○	○	○	○
Watching TV (टि.भी हेर्दा)	○	○	○	○
Sitting inactive in a public place (e.g., a theater or meeting (सार्वजनिक स्थानमा निष्क्रिय भएर बस्दा (जस्तै, थिएटर वा बैठक)	○	○	○	○

	No chance of dozing (निदाउन सम्भावना छैन)	Slight chance of dozing (निद्रा लाग्ने अलिकति सम्भावना छ)	Moderate chance of dozing (निद्रा लाग्ने मध्यम सम्भावना छ)	High chance of dozing (निदाउने उच्च सम्भावना छ)
As a passenger in a car for an hour without a break(यात्रीको रूपमा कारमा ब्रेक बिना एक घण्टाको लागि यात्रा गर्दा)	○	○	○	○
Lying down to rest in the afternoon when circumstances permit (परिस्थिति अनुकलाता मिल्दा दिउँसो आराम गर्न सुत्ने वा पल्टिने गर्छु)	○	○	○	○
Sitting and talking to someone (कसैसँग बसेर कुरा गर्दा)	○	○	○	○
Sitting quietly after a lunch without alcohol (रक्सी बिना दिउँसोको खाना खाएपछि चुपचाप बस्था)	○	○	○	○
In a car, while stopped for a few minutes in traffic (ट्राफिकमा गाडी केही मिनेट रोकिएको बेला)	○	○	○	○

Health-Related Quality of Life स्वास्थ्य जीबन गुणस्तर

The next set of questions asks for your views about your health. This information will help keep track of how you feel and how well you are able to do your usual activities:
यो प्रश्नावलीले तपाईंको स्वास्थ प्रति तपाईं विचार कस्तो छ भनी प्रश्न गर्छ। यस किसिमको जानकारीले तपाईं कस्तो महसुस गर्नुहुन्छ र आफ्नो दैनिक गतिविधि गर्न कतिको सक्षम हुनुहुन्छ भन्ने कुरा दर्शाउँछ।

In general, would you say your health is:
सामान्यतया, तपाई आफनो स्वास्थ्य कस्तो छ भन्नुहुन्छ?

- ○ Excellent (सर्वोत्तम)
- ○ Very good (अति उत्तम)
- ○ Good (उत्तम)
- ○ Fair (ठिकै)
- ○ Poor (अति खराब)

The following questions are about activities you might do during a typical day. Does your health now limit you in these activities? If so, how much?
तलका प्रश्नहरु एक दिनमा गर्न सक्ने साधारणतया गतिविधिहरुका बारेमा छन् । के तपाईंको स्वास्थ्यले अहिले यी गतिविधिहरु गर्न बाधा पुर्याउने गरेको छ?

	Yes, limited a lot (हो, धेरै बाधा पुऱ्याएको छ।)	Yes, limited a little (हो, केही बाधा पुऱ्याएको छ।)	No, not limited at all (होइन, कुनै पनि बाधा पुऱ्याएको छैन।)
Moderate activities, such as moving a table, pushing a vacuum cleaner, bowling or playing golf (मध्यम गतिविधिहरु जस्तै टेबुल सार्न, भुइँ बढार्न, बारीमा काम गर्न वा साइकल चलाउन)	○	○	○
Climbing several flights of stairs (अनेक खुड्किला वा भर्याङ्. चड्ने)	○	○	○

During the past 4 weeks, have you had any of the following problems with your work or other regular daily activities as a result of any emotional problems (such as feeling depressed or anxious)?
पछिल्लो चार हप्तामा तपाईका भावनात्मक समस्याहरुको कारण जस्तै निराश वा चिन्तित भएर तपाईको काम वा अरु दैनिक गतिविधिमा निम्न लिखित समस्या आए?

	Yes (हो)	No (होइन)
Accomplished less than you would like (चाहे भन्दा कम काम सक्नु भयो)	○	○
Did work or activities less carefully than usual (काम वा अरु गतिविधिहरु सामान्य भन्दा कम होश पुऱ्याएर गर्नुभयो)	○	○

During the past 4 weeks, how much pain interferes with your normal work (including work outside the home and housework)?

पछिल्लो चार हप्तामा, दुखाईले तपाईको सामान्य काममा (घर भित्र र बाहिर दुवै काम गरी) कति मात्रामा बाधा पुऱ्यायको?

- Not at all (कहिले भएन)
- A little bit (कहिले कहिँ)
- Moderately (केहि मात्रामा)
- Quite a bit (अलि धेरै)
- Extremely (एकदम धेरै)

These questions are about how you have been feeling during the past 4 weeks. For each question, please give the one answer that comes closest to the way you have been feeling. How much of the time during the past 4 weeks...
यी प्रश्नहरु तपाईले कस्तो महसुस गर्नुहुन्छ र पछिल्लो चार हप्ता तपाईका लागि कस्ता रहे भन्ने बारे हो। हरेक प्रश्नको लागि तपाईंले महसुस गर्नु भएको सबै भन्दा नजिकको एक उत्तर दिनुहोस्। कति पटक पछिल्लो चार साता

	All of the time (हरेक पल्ट)	Most of the time (धेरै जसो)	A good bit of the time (केहि पल्ट)	Some of the time (कहिलेकाहीँ)	A little of the time (थोरै समय)	None of the time (कहिले पनि भएन)
Have you felt calm and peaceful? (तपाई शान्त र निश्चिन्त रहनु भयो ?)	O	O	O	O	O	O
Did you have a lot of energy? (तपाई स्फुर्त रहनु भयो ?)	O	O	O	O	O	O
Have you felt down-hearted and blue? तपाई हतोत्साहित र उदास हुनुभयो ?	O	O	O	O	O	O

During the past 4 weeks, how much of the time have your physical health or emotional problems interfered with your social activities (like visiting friends, relatives, etc.)?
पछिल्लो चार हप्तामा तपाईको शारीरिक स्वास्थ्य वा भावनात्मक समस्याहरुका कारण तपाईको सामाजिक क्रियाकलाप (जस्तै साथीहरु, आफन्तहरु भेट्न जाने आदि) मा कुन हद् सम्म वाधा पुराएको छ?

- All of the time (हरेक पल्ट)
- Most of the time (धेरै जसो)

- Some of the time (केहि पल्ट)
- A little of the time (कहिले कहिँ)
- None of the time (कहिले पनि भएन)

Monkey Pox

What kind of disease does monkeypox cause?
Monkeypox ले कस्तो प्रकारको रोग लाग्दछ?

- Chronic disease (दीर्घ रोग)
- Immune disease (प्रतिरक्षा सम्बन्धी रोग)
- Infectious disease (संक्रामक रोग)
- Hereditary (वंशाणुगत)
- Inflammation (सूजन वा सुन्निने)
- Metabolic (मेटाबोलिक)

Monkeypox is a new infection that appeared this year 2022.
Monkeypox २०२२ मा देखापरेको एक नयाँ संक्रमण हो।

- No (होइन)
- Yes (हो)
- I don't know (थाहा छैन)

Monkeypox is a sexually transmitted infection.
Monkeypox यौन सम्पर्कबाट सर्ने एक रोग हो।

- No (होइन)
- Yes (हो)
- I don't know (थाहा छैन)

Chickenpox and monkeypox are the same disease.
Chickenpox र Monkeypox रोगहरू एउटै हुन्।

- No (होइन)

- Yes (हो)
- I don’t know (थाहा छैन)

Monkeypox is common in South Asian Countries?
दक्षिण एसियाली देशहरूमा Monkeypox प्रचलित छ?

- No (छैन)
- Yes (छ)
- I don’t know (थाहा छैन)

Monkeypox is common in West and Central African countries.
Monkeypox पश्चिम र मध्य अफ्रिकी देशहरूमा प्रचलित छ।

- No (होइन)
- Yes (हो)
- I don’t know (थाहा छैन)

There are many cases recorded in Nepal.
नेपालमा धेरै Monkeypoxका केसहरू रेकर्ड भएका छन्।

- No (होइन)
- Yes (हो)
- I don’t know (थाहा छैन)

Monkeypox cases are increasing in the USA and Europe.
संयुक्त राज्य अमेरिका र युरोपमा Monkeypox को केसहरू बढ्दै छन्।

- No (होइन)
- Yes (हो)
- I don’t know (थाहा छैन)

Monkeypox is a contagious viral disease.
Monkeypox संक्रामक भाइरल रोग हो।

- No (होइन)
- Yes (हो)
- I don't know (थाहा छैन)

Monkeypox is a contagious bacterial disease.
Monkeypox संक्रामक जीवाणुबाट सर्ने (bacterial) रोग हो।

- No (होइन)
- Yes (हो)
- I don't know (थाहा छैन)

Monkeypox is easily transmitted from one person to another.
एक व्यक्तिबाट अर्को व्यक्तिमा Monkeypox सजिलै सर्छ।

- No (होइन)
- Yes (हो)
- I don't know (थाहा छैन)

Monkeypox is transmitted to humans through the bites and scratches from infected animal.
संक्रमित जनावरको टोकाइबाट र खरोंचहरूबाट (चिथारेर) Monkeypox मानिसमा सर्छ।

- No (होइन)
- Yes (हो)
- I don't know (थाहा छैन)

People with monkeypox can transmit the disease to others (the disease is transmitted between humans).
Monkeypox भएका मानिसहरू देखि अरू मानिसलाई सार्न सक्छन् (यो रोग एक व्यक्तिबाट अर्को व्यक्तिमा सर्छ)।

- No (होइन)
- Yes (हो)
- I don't know (थाहा छैन)

Monkeypox is spread by droplets (coughing and sneezing).
थुक वा खकारबाट खोक्दा र हाच्छिउँ गर्दा Monkeypox सर्छ।

- No (होइन)
- Yes (हो)
- I don’t know (थाहा छैन)

The first symptoms of monkeypox are like the flu.
Monkeypox को पहिलो लक्षणहरू रुघा खोकी (फ्लु) जस्तै हुन्छन् ।

- No (होइन)
- Yes (हो)
- I don’t know (थाहा छैन)

Skin rash is a symptom of monkeypox.
छालामा डाबर वा खटिराहरू Monkeypox को एक लक्षण हो।

- No (होइन)
- Yes (हो)
- I don’t know (थाहा छैन)

Monkeypox only affects males.
Monkeypox ले पुरुषलाई मात्र असर गर्छ।

- No (होइन)
- Yes (हो)
- I don’t know (थाहा छैन)

Hand sanitizers and face masks are important in preventing monkeypox.
Monkeypox बाट बच्नको लागि ह्यान्ड सेनिटाइजर र फेस मास्कहरू महत्त्वपूर्ण छन्।

- No (होइन)
- Yes (हो)

- I don't know (थाहा छैन)

There is a special treatment for monkeypox.
Monkeypox को लागि विशेष उपचार छ।

- No (होइन)
- Yes (हो)
- I don't know (थाहा छैन)

Monkeypox is spread through bodily fluids.
Monkeypox शारीरिक तरल पदार्थ को माध्यम बाट फैलन्छ।

- No (होइन)
- Yes (हो)
- I don't know (थाहा छैन)

There is a monkeypox vaccine available in Nepal.
नेपालमा Monkeypox को खोप उपलब्ध छ।

- No (होइन)
- Yes (हो)
- I don't know (थाहा छैन)

The chickenpox vaccine I got in childhood protects me from monkeypox.
मैले बाल्यकालमा पाएको ठेउला (Chickenpox) को खोपले मलाई Monkeypox पोक्सबाट जोगाउँछ।

- No (होइन)
- Yes (हो)
- I don't know (थाहा छैन)

There is a smallpox vaccine that can be used for monkeypox.
Monkeypox को लागि बिफर (smallpox) खोप प्रयोग गर्न सकिन्छ।

- No (होइन)

- Yes (हो)
- I don’t know (थाहा छैन)

Powered by Qualtrics
