# Supplementary material for: Mediating role of food insecurity in the relationship between perceived MSM related stigma and depressive symptoms among men who have sex with men in Nepal
Source: PLoS One. 2024 Jan 2;19(1):e0296097. doi: 10.1371/journal.pone.0296097 (PMC10760890; doi:10.1371/journal.pone.0296097)
Supplement: Attachment — Submitted filename: Reviewer comments - MSM Nepal stigma depressive symptoms FI.docx [file pone.0296097.s002.docx]

Overall, I congratulate the authors on this interesting contribution to understanding food insecurity’s role as a mediating factor between stigma and depressive symptoms in MSM. The core arguments are clear and well-aligned, and the statistical tests are appropriate for the sample. The authors have done a nice job of presenting simple and easily understandable figures, though the readability of the tables could be improved. Though most language is good, I recommend an overall proofread before considering direct publication because there are a few errors which significantly influence interpretation. I recommend this article for publication after minor revisions. My detailed comments are below:

| Section | Comments |
| --- | --- |
| all | Please check style throughout to avoid incomplete statements, mixed word order, and apparent typos which impact understanding,  e.g., in Abstract, “The impact of **food stigma** on depressive symptoms […]”  e.g. in Measures, “[…] educational status **(you mean level?)**, monthly income […]”  e.g. in Statistical analysis, “[…] mediating effects of **medication** on these outcomes.” |
| Measures – Food insecurity | Mention a few key details about the FIES as used, namely 1) was the reference period 30 days or 12 months?, 2) given the sample size and type, clarify to what extent unadjusted raw score was used vs. applying infit/outfit/etc. measures?, and 3) not necessary everywhere, but here in measures please specify that the concept of “food insecurity” in this paper appears to be equivalent with “moderate to severe food insecurity” according to the FIES |
| Measures – Stigma | Please indicate if any cut-off values were used to classify stigma, and if so, how these relate or were adapted from previous literature. |
| Results | “Participants who are gay had higher stigma than their bisexual counterparts”  This is the first time the terms “gay” and “bisexual” are used. If you want to use them, please define them before the Results section by linking them clearly to the term “MSM” |
| Results | Given the potentially sensitive nature of these questions, I suggest noting levels of non-response. Related to that, you could indicate n in Table 2, to keep the general sample size in mind but also note by item (if relevant) any higher non-response to certain items as compared to others. |
| Results – Table 2 | It’s not immediately clear why the bolded values are bolded. Make a footnote or explicitly address each one in the text. |
| Results - Mediation of association between stigma and depressive symptoms by food insecurity | Clarify if the result stated in the text comes from Model 1 or Model 2. Unless I’m misunderstanding, Table 3 shows the that 20.6% comes from Model 2, but this seems contradicted by the text just before the table. |
| Results | I find myself wondering about the “socio-demographic” variable of income being used as an adjustment variable. Income would be very strongly linked with food insecurity, especially as measured by the FIES. How did the authors consider this relationship in the analysis and discussion? |
| Discussion | “Our observed prevalence of food insecurity (29.2%) was higher than that of other studies reported among MSM (16) and other sexual minorities like transgender, female sex workers (9). Evidently, food insecurity among Nepali at the national level is comparatively higher than that of other countries, because Nepal is predominantly reliant on agriculture and is facing a growing trade deficit (17).”  I’d suggest caution when making conclusions about this which make assumptions about the entire population, as you do in the second sentence. Have you considered how your sampling strategy as compared with other studies may have affected these values? |
| Discussion | Broadly, the Discussion would benefit from consideration of depression as a potential contributor to food insecurity and stigma, as opposed to only exploring the other direction. Throughout the introduction, modeling, and limitations the multi-directionality of these pathways is considered, but in my view it’s not well-addressed in the discussion. |
| Discussion | “Additionally, individuals who face food insecurity may also be more likely to engage in risky activities, such as sex work, in order to secure food and other essentials (31).”  Suggest incorporating the term “coping strategies” to describe actions like these, to benefit the authors in future literature searches around this developing topic. |
| Discussion – Limitations | “Lastly, the study did not collect data on other forms of food insecurity, such as poverty-related food insecurity, which limits our ability to fully understand the role of food insecurity in the relationship between stigma and depression.”  I disagree with this statement as I understand it. The FIES questions explicitly explore “poverty-related” food insecurity (most food insecurity could be described that way). I’d remove this statement |
